# Changes in the Education-Health Gradient Within U.S. States, 1993–2019

**DOI:** 10.1007/s11113-026-10020-8

**Published:** 2026-06-22

**Authors:** Jennifer Karas Montez, Iliya Gutin, Julia M. Finan, Anna Zajacova, Scott Landes

**Affiliations:** 1 Syracuse University, Syracuse, USA; 2 Western University, London, Canada

**Keywords:** Health, Education, Gradient, U.S. States, Disparities

## Abstract

The association between educational attainment and health (i.e., the education-health gradient) among U.S. adults has strengthened in recent decades; however, explanations for this trend are incomplete. This study builds on recent work showing that the strength of the gradient differs across U.S. states. We asked, how and why has the gradient changed over time in each U.S. state? Using 27 years of cross-sectional data from the 1993–2019 Behavioral Risk Factor Surveillance System on adults ages 30–64 years, we estimated how the association between educational attainment and self-rated health changed over time in each state. We also assessed the contribution of six individual-level factors, such as adults’ income, and three state-level contextual factors, such educational composition and policy context, to changes in the association in each state. The gradient increased significantly in 49 of the 50 states during the period, but the magnitude of the increase differed notably by state. In most states, the largest single contributor to the increasing gradient was household income. Adults’ employment and obesity also contributed to the increasing gradient to varying degrees in many states, while smoking suppressed the increase in the gradient in over half of states. The state-level contextual factors contributed little to the increasing gradient. The findings underscore the value of a subnational focus for explaining and potentially reversing the growth in the education-health gradient.

**Supplementary Information:**

The online version contains supplementary material available at 10.1007/s11113-026-10020-8.

## Introduction

Educational attainment is positively associated with health and longevity in the United States. Because the association holds across all levels of educational attainment, whether measured by years of education or by formal credentials, it is often described as a gradient. Importantly, the education-health gradient has grown stronger over time. The strengthening gradient has been extensively documented, but explanations for it remain incomplete. The current study aims to advance explanations by leveraging geographic heterogeneity in the gradient, building on a growing number of studies showing that the gradient is stronger in some U.S. states than others. Specifically, the current study evaluates whether and why the association between educational attainment and self-rated health (i.e., the education-health gradient) among adults ages 30–64 has changed over time within each of the 50 U.S. states. It first estimates how the gradient changed across a 27-year period, from 1993 to 2019, in each state. It then estimates the contribution of nine factors to the changing gradient in each state. Six are individual-level factors thought to be key mechanisms through which educational attainment affects health: adults’ income, employment, healthcare, marital status, smoking, and obesity. Three are state-level contextual factors that have changed over time in ways that may have affected the gradient. These include states’ educational composition, share of immigrants, and overarching policy contexts. The findings underscore the value of a subnational focus for explaining and potentially reversing growth in the education-health gradient.

## Background

Educational attainment, whether measured by years of education or formal credentials, is a robust predictor of adult health and longevity in the United States (Montez & Bisesti, 2024). Higher educational attainment is associated with lower probabilities of hypertension (CDC, 2020), diabetes (CDC, 2022), depressive symptomology (Bauldry, 2015), cardiovascular disease (Kubota et al., 2017), functional limitations (Zajacova & Montez, 2017), and having multiple chronic conditions (Johnson-Lawrence et al., 2017). Adults with higher educational attainment live longer (Sasson & Hayward, 2019) and spend a greater proportion of their years in good health (Crimmins et al., 2018). For example, among U.S. women who survived to age 65, those without a high school credential (neither diploma nor GED) could expect to live 32% of their remaining years without pain, while those with at least a bachelor’s degree could expect to live 45% of remaining years without pain (Sun et al., 2023). In this study, we refer to the positive association between educational attainment and health-related outcomes as the education-health association, education-health gradient, or simply the gradient.

Several conceptual frameworks help explain the existence of the gradient. For instance, the Human Capital (Becker, 2009) and Learned Effectiveness (Mirowsky & Ross, 2003) frameworks assert that education changes individuals in ways that ultimately benefit their health. Specifically, through schooling, people develop cognitive and non-cognitive skills and a sense of personal control, which they then use to garner health-related advantages, such as income, and avoid health-related risks, such as smoking. A prominent framework for understanding the education-health gradient is Fundamental Cause Theory (FCT, Clouston & Link, 2021; Link & Phelan, 1995). According to FCT, the gradient emerges in times and places where salubrious resources, such as money and social ties, become disproportionately allocated to individuals with higher educational attainment. Illustrating the time dimension of FCT, Link (2008) showed that, following the 1964 U.S. Surgeon General’s report, knowledge about the health risks of smoking spread most rapidly among individuals with higher educational attainment and, consequently, a strong negative association between educational attainment and smoking emerged. Illustrating the place dimension of FCT, Mackenbach et al. (2015) found that the association between educational attainment and mortality from preventable causes of death was stronger in Central and Eastern European countries, where inequalities in material resources are larger, than in Nordic countries, where those inequalities are smaller.

Grounded in FCT, studies have revealed that educational attainment shapes health through myriad individual-level mechanisms, which are often grouped into economic conditions, psychosocial resources, health behaviors, and healthcare (Cutler & Lleras-Muney, 2010; Hummer & Lariscy, 2011; Mirowsky & Ross, 2003). Individuals with higher educational attainment tend to have: favorable economic conditions such as stable employment and higher incomes; psychosocial resources such as stable marriages and robust friendship networks; health-promoting behaviors such as physical activity and avoidance of health-damaging behaviors such as smoking; and access to quality healthcare (Cutler & Lleras-Muney, 2008; Hummer & Lariscy, 2011; Mirowsky & Ross, 2003). Factors such as these are sometimes referred to as indirect mechanisms based on the assumption that one’s educational attainment shapes access to them, which, in turn, affects one’s health. As an example, a study using U.S. data from the 1990s estimated that one-third of the association between adults’ educational attainment and mortality risk operated through economic conditions, particularly income, and another 30% operated through behaviors such as smoking (Cutler & Lleras-Muney, 2010).

## The Gradient Becomes Stronger

Since the 1960s, educational attainment has become an increasingly strong predictor of health-related outcomes in the United States (see Montez & Bisesti, 2024). Between the 1960s and 1980s, the gradient grew stronger mainly because health and longevity improved most for adults with higher educational attainment. The story is different starting in the 1980s. Many studies find that, since the 1980s, the gradient grew stronger mainly because the health and longevity of adults with higher educational attainment, particularly those with at least a bachelor’s degree, continued to improve while the health and longevity of adults with lower educational attainment, such as those with a high school credential or less, stagnated or declined (Case & Deaton, 2015; Meara et al., 2008; Miech et al., 2011; Montez et al., 2011; Sasson, 2016; Sasson & Hayward, 2019). The growth in the gradient has been striking. Educational attainment has become one of the strongest predictors of adult health and longevity in the United States (Montez & Bisesti, 2024).

Although the gradient has become stronger, explanations for the trend are less clear. Fundamental cause theory posits that the gradient becomes stronger when health-related resources and risks become more unequal across educational attainment. This can occur for myriad reasons. For example, new medical technologies and preventive strategies for certain diseases can strengthen the gradient when access to those new resources becomes concentrated among individuals with higher educational attainment (Masters et al., 2015). Another example is the rise in opioid misuse and deaths. Consistent with FCT, the rise primarily occurred among individuals with lower educational attainment, which exacerbated the gradient (Ho, 2017). In addition, some scholars speculate that structural forces in recent decades—including deindustrialization, globalization, declines of labor unions, rising income inequality, and a policy environment that increasingly favors corporations over workers—have strengthened the gradient by disproportionately harming the economic and psychosocial wellbeing of individuals with lower educational attainment (e.g., Case & Deaton, 2020; Montez et al., 2021; Venkataramani et al., 2020). Common to all these examples is the FCT proposition that educational attainment has become more critical for accessing health-related resources and avoiding health-related risks.

The current study examines whether six individual-level factors may have contributed to the increasing education-health gradient in recent decades. We selected these six factors for several reasons. They span the main groups of mechanisms thought to explain the gradient (economic conditions, psychosocial resources, health behaviors, and healthcare), they are widely understood as key contributors to the strengthening gradient (e.g., Montez & Zajacova, 2013), and they are available in the dataset for this study. The six individual-level factors include income, employment, healthcare coverage, smoking, obesity, and marital status. We next describe each briefly below.

Major structural changes in the labor market since the 1980s have disproportionately benefitted the economic wellbeing of individuals with higher educational attainment, especially those with at least a bachelor’s degree, but gutted opportunities among others for stable employment, livable wages, and access to quality healthcare (Case & Deaton, 2020; Lynch, 2006; Montez & Zajacova, 2013). Other evidence implicates health behaviors as an explanation for the increasing gradient. For example, declines in cigarette smoking have disproportionately occurred among adults with higher educational attainment (Link, 2008). In addition, the prevalence of obesity, which can be a marker of both health behaviors and metabolic functioning, has risen across educational attainment levels, but the rise has been more pronounced among those without a bachelor’s degree, especially among women (Ogden et al., 2010). Psychosocial resources have also become more strongly patterned by educational attainment in recent decades. For example, educational attainment has become a stronger predictor of marital status (Parker & Stepler, 2017). Although these six individual-level factors appear to have contributed to the increasing gradient to various degrees, explanations for the increase remain incomplete (e.g., Cutler & Lleras-Muney, 2010; Lynch, 2006; Montez & Zajacova, 2013).

### Advancing Explanations for the Changing Gradient

This study aims to advance explanations in two main ways. First, in addition to examining the six individual-level factors described previously, it examines three contextual-level factors that may have contributed to the strengthening gradient, including changes in population composition and policy contexts. Some researchers have asserted that the education-health gradient has increased primarily because the composition of the population with certain educational attainment levels has changed over time, not because educational attainment itself has become more important for health-related risks and resources (e.g., Bound et al., 2015; Hendi, 2015). One such assertion is that the rise in educational attainment over time means that people with low educational attainment in recent years are different than their same-educated peers in prior decades—they may have had early-life health issues, behavioral problems, or other early-life health risks that cut short their educational attainment. As this group becomes smaller in size and more disadvantaged over time, this could exacerbate the gradient by making the health of adults with low educational attainment appear to decline over time. This hypothesis has been partially supported by some studies (Bound et al., 2015; Hendi, 2015) but not others (Case & Deaton, 2025; Frase & Bauldry, 2022). The current study includes an annual measure of the share of college graduates in each state as a contextual factor.

Changes in the share of immigrants in the population may also have affected the gradient, but the direction of that effect is unclear. On the one hand, educational attainment among immigrants has risen in recent decades (Krogstad & Radford, 2018) and immigrants generally exhibit better health and health behaviors compared with U.S.-born individuals (Hamilton & Hagos, 2020). This could strengthen the gradient by disproportionately improving the average health of people with higher educational attainment. On the other hand, educational attainment tends to be less beneficial for the health of immigrants than it is for U.S.-born individuals (Assari et al., 2020), so any improvement may be small. The current study includes an annual measure of share of immigrants as the second contextual factor.

The third contextual factor is policy contexts. The U.S. policy context has changed in recent decades, particularly at the U.S. state level, with many states moving their policy contexts toward either a more liberal or conservative direction (Grumbach, 2018). For example, some states have raised the minimum wage, enacted an earned income tax credit, expanded Medicaid, and increased tobacco taxes (e.g., Dow et al., 2020; Friedson et al., 2023; Kaufman et al., 2020; Kravitz-Wirtz et al., 2020; Montez & Grumbach, 2023; Sharkey & Kang, 2023). Many other states have either not implemented or scaled back these health-promoting policies. Some, for example, have enacted so-called right to work laws that dismantle the power of labor unions, and some states have rolled back the number of firearm safety laws (Montez & Grumbach, 2023). Many such policies are disproportionately salient for persons with low income or educational attainment. Therefore, the major changes in states’ policy contexts in recent decades may have affected the gradient.

These state-level contextual changes may have impinged on the economic, psychosocial, and behavioral pathways between education and health. For instance, states that raised their minimum wage or increased cigarette taxes (Riley, 2024) may have weakened the pathway from education to income to health. States that enacted right to work laws may have strengthened the pathway from education to employment and income to health. Changes in states’ policy contexts may have affected the education-health association through additional, unknown pathways. In sum, because the policy contexts of U.S. states impact population health overall and perhaps most keenly among adults with low educational attainment, changes in those contexts in recent decades may have affected the education-health gradient.

To capture changes in states’ policy contexts over time, the current study uses an annual summary measure of the ideological orientation of 148 specific state policies on a liberal-to-conservative continuum (Caughey & Warshaw, 2016). This holistic and validated measure captures the variation in policy contexts between states and over time. Studies using this measure find that conservative policy contexts are associated with worse health-related outcomes (Zacher et al., 2025). We use this measure instead of specific policies for conceptual and empirical reasons. The ideological orientation of policies within states has become increasingly aligned, reflecting a broader polarization in which states policy orientations have moved toward either the left of right end of the political spectrum. Consequently, states can increasingly be characterized by a holistic set of policies that are largely salubrious or detrimental for health. Using a single, validated summary measure allows us to capture these overall changes and avoid making assumptions about which specific policy or subset of policies is most important.

### Leveraging Differences in the Gradient Across U.S. States

 The second way that we advance explanations is to examine changes in the gradient at the U.S. state level rather than the national level. This approach builds on recent cross-sectional studies that have examined the education–health gradient across U.S. states, and extends them by analyzing how the gradient has changed over time within each state. The rationale is that differences across states in the gradient trends over time can provide new insights about factors that shape the gradient. For example, if the association is strongest in states where the gap in smoking between adults higher versus lower educational attainment is largest, then state policies on tobacco consumption may be a key explanation.

These studies have found that the magnitude of the gradient differs markedly across states for several outcomes such as self-rated health, pain, functional limitations, disability, and mortality (Huang et al. 2023; Kemp et al. 2022; Montez et al. 2017, 2019a, b). Recent work has started to investigate why the magnitude of the gradient differs. One study found that the magnitude differs across states mainly because health-related outcomes of adults with lower educational attainment differs across states (Montez et al., 2017). Another study documented the association between education and self-rated health among working-age adults in each state and found that the association was strongest in states where one’s educational attainment level was an especially strong pathway to employment and income (Montez & Cheng, 2022). This can occur if, for example, states’ job markets, labor policies such as minimum wage levels, and economic safety nets exacerbate the importance of educational attainment for obtaining stable employment and livable wages. We build on these studies showing that the education-health gradient differs across states to examine how and why the gradient has *changed over time* within states.

## Aims

This study seeks to better understand how the education-health gradient among working-age adults has changed over time within U.S. states. It has two main aims. The first aim is to estimate how the gradient changed during the 1993 to 2019 period among working-age adults in each state. The second aim is to estimate how much of the change in the gradient in each state can be accounted for by six individual-level factors (income, employment, healthcare coverage, marital status, smoking, and obesity) and three state-level contextual factors (states’ share of college graduates and immigrants, and states’ policy contexts) hypothesized in the Background section.

We focus on self-rated health for four reasons. For one, the association between educational attainment and self-rated health is strong (Zajacova et al., 2012). Second, the association between educational attainment and self-rated health among U.S. adults has increased over time, at least for some age groups and birth cohorts (Goesling, 2007; Liu & Hummer, 2008; Lynch, 2006; Schellekens & Ziv, 2020). Third, the strength of the association differs across states (Montez et al. 2019a). Fourth, self-rated health does not require a medical diagnosis, which could bias the results given disparities in health insurance by education level.

We focus on working-age adults because their health and mortality trends have been troubling in recent decades (Case & Deaton, 2015; NASEM, 2021). This focus is also informed by methodological and conceptual considerations. First, setting the lower threshold at 30 years of age helps capture completed education through a bachelor’s degree for most respondents, and follows prior studies of the association between education and self-rated health (Goesling, 2007; Zajacova et al., 2012). Second, our inclusion of employment, income, and healthcare coverage as potential explanations for trends in the association is mostly relevant for working-age adults.

## Data and Methods

### Analytic Sample

Our analyses used 27 years of data, spanning 1993 through 2019, from the Behavioral Risk Factor Surveillance System (BRFSS, CDC, 2023). The BRFSS is an annual, cross-sectional survey of noninstitutionalized adults, with data representative at the state level. The BRFSS was a landline telephone survey until 2011 when it began using both landline and cellphone-only sampling frames. We start with the 1993 BRFSS because it is the first year that all 50 states were included, and we end with 2019 to avoid data concerns during COVID-19. We note that BRFSS does not contain data for 1993 in Wyoming, 1993 or 1994 data in Rhode Island, 2004 data in Hawaii, and 2019 data in New Jersey.

The BRFSS sample across the 1993–2019 period included 4,976,964 adults 30–64 years of age in the 50 states. Among those respondents, 4,904,293 had complete data on the variables for Aim 1. The 1.5% with incomplete data included 0.3% missing self-rated health, 0.2% missing education, 1.0% missing race-ethnicity, and < 0.1% missing sex. We used this analytic sample for Aim 1. To create the analytic sample for Aim 2, we first excluded the 1993 BRFSS because its income categories were not harmonized with other years (1.2% of respondents). We then excluded 8.4% of respondents who were unable to work, which we explain later, and lastly dropped 14.7% of respondents missing data on one or more individual-level factor. This included 7.8% missing income, 3.4% missing obesity, 1.0% missing both income and obesity, and < 1% missing on one or more of the other factors. The final sample size for Aim 2 is 3,783,645 adults.

## Measures

### Self-rated Health

The BRFSS asked respondents, “Would you say that in general your health is excellent, very good, good, fair, or poor?” We dichotomized the responses into favorable health (which includes excellent, very good, and good) and unfavorable health (which includes fair or poor). Dichotomization facilitates interpretation of the model coefficients and increases test-retest reliability (Zajacova & Dowd, 2011), with the favorable-unfavorable dichotomization providing more valid and reliable estimates than higher cut-points (Plante et al., 2024). Prior work has found that the dichotomized and original ordinal specifications provide similar findings when predicting self-rated health from sociodemographic covariates (Manor et al., 2000).

### Educational Attainment

The BRFSS asked respondents to report the highest completed grade or year of school. The survey captures responses in six categories. We converted the responses into a semi-continuous measure by assigning an approximate year of completed education to the midpoint of each category, following other studies (e.g., Hayward et al., 2015). Adults who stated they never attended school or only completed kindergarten were assigned zero years of schooling, adults who completed grades one through eight were assigned 4.5 years of schooling, adults who completed grades nine through 11 were assigned 10 years of schooling, adults who completed grade 12 or received a GED were assigned 12 years of schooling, adults who completed one to three years of college were assigned 14 years of schooling, and adults who completed four years of college or more were assigned 18 years. This operationalization is best suited for addressing the study’s aims. As explained in the analytic strategy below, it allows for a single, interpretable, and comparable estimate of how the education-health gradient changed over time in each state.

### Covariates

Age is included as a categorical variable for five-year age groups from 30 to 34 to 60–64 years. The survey provides sex as female or male. It provides seven response categories for the question, “What is your race?” Because the measure must be used in all state-stratified models, and some states had negligible numbers of several minoritized groups, we collapsed the responses into non-Hispanic White and other. Lastly, we included an indicator of whether the respondent was from the landline or cellphone-only sample frame (see Blanchflower & Oswald, 2020).

### Nine Hypothesized Factors

For Aim 2, we examined the contribution of nine factors, described in the Background, to the changing gradient in each state. We selected six individual-level factors that have long been considered mediators between educational attainment and health, including income, employment, healthcare, marital status, smoking, and obesity. We also included three state-level contextual factors that have more recently been hypothesized to matter. These included states’ shares of college graduates and immigrants, and states’ policy context.

To assess annual *household income*, respondents were asked a series of questions about their total household income from all sources. The survey provides the responses as categories, which we converted into a semi-continuous measure using the recommended upper bound method (Hest, 2019). For example, the household income category of “less than $10,000” was converted to $10,000, the household income category of “$10,000 to $14,999” was assigned $15,000, and so on, through $100,000. To assess *healthcare coverage*, we used the yes-no responses to the following question, “Do you have any kind of health care coverage, including health insurance, prepaid plans such as HMOs, or government plans such as Medicare?”

To assess *employment status*, the survey asks, “Are you currently employed for wages, self-employed, out of work, a homemaker, student, retired, or unable to work?” We excluded respondents for Aim 2 if they reported that they were unable to work, because this employment category is largely endogenous with our outcome variable. Among those who were unable to work, 69.9% were in fair or poor health, versus just 9.0% of employed respondents (Online Supplement Table S1 contains more information on these adults). If being unable to work is a proxy for unfavorable health, then including it as a mediator in the employment variable is problematic. Among adults who were able to work, we dichotomized employment into employed (for wages or self-employed) and not employed (out of work, homemaker, student, or retired).

Despite the many strengths of the BRFSS for our study, it does not contain measures of psychosocial resources such as self-esteem or optimism. However, it does contain information on marital status which we use as a relatively crude indicator of social support as a type of psychosocial resource. We included an indicator of *marital status*, distinguishing currently married versus nonmarried. We dichotomized it because the age-adjusted percentage of adults reporting favorable health was similar across nonmarried statuses (ranging from 74.9% to 79.2%) and notably lower than the 87.9% for married adults. To capture behavioral mechanisms, we included cigarette *smoking* and *obesity*, recognizing that the latter can reflect behaviors and metabolic conditions. Smoking is a dichotomous measure, distinguishing current smokers versus never and former smokers. The age-adjusted percentage of adults reporting favorable health is notably worse among current smokers (73.4%) than never (87.8%) and former (83.5%) smokers. The obesity variable was coded as 1 if the respondents’ body mass index (BMI) was 30.0 or higher. The BRFSS calculated respondents’ BMIs based on self-reported height and weight.

As described in the Background, changes in the population composition across educational attainment levels may also underlie changes in the education-health gradient. The BRFSS does not provide respondents’ immigration status, so we included the annual percentage of each state’s population who were *immigrants*. This data were provided by the Migration Policy Institute (2024) for 1990, 2000, 2010, and 2019. We used linear interpolation for intra-decade years. We also included the annual percentage of working-age adults with at least a bachelor’s degree in each state, estimated from the BRFSS using the sample weights.

Lastly, we included a summary measure of state policy contexts. We used an annual index that summarizes the ideological orientation of 148 state policies on a liberal-to-conservative continuum. It was created by Caughey and Warshaw (2016) using dynamic Bayesian factor analysis. Its average is 0 and it ranges from − 2.9 to 3.5. This policy index is a strong predictor of population health (e.g., Zacher et al., 2025). The 148 policies within the index represent the “salient policy activities of U.S. states” across major domains such as abortion, civil rights, criminal justice, environment, gun safety, labor, and taxes (Caughey & Warshaw, 2016). The index creators showed that the 148 policies can be collapsed into a single measure that adequately explains policy variation across states and exhibits convergent and construct validity.

### Approach

To address Aim 1, we estimated linear probability models stratified by U.S. state. We chose these models given our focus on interaction terms in the models and the challenges in interpreting and decomposing interactions in nonlinear models (e.g., Mize, 2019). The general form of the model is shown in Eq. 1, where $\:{Y}_{i}$ = 1 if individual *i* reported favorable health and 0 otherwise. Education, denoted as “educ” in the equation, and year are continuous measures, with year scaled from 0 (calendar year 1993) to 1 (calendar year 2019). The coefficient vector $\:{\beta\:}_{c}$ includes the four covariates listed above. The estimand of interest is the interaction coefficient $\:{\beta\:}_{3}$. A positive interaction would imply that adults with higher educational attainment experienced greater improvements in self-rated health during the period than did adults with lower educational attainment and that the gradient increased over time. All models used robust standard errors because of the binary *Y* and were estimated with Stata MP 18.0.

1 $$\begin{aligned} {\mathrm{Y}}_{{\mathrm{i}}} =\, & \beta _{0} + \beta _{1} {\mathrm{educ}}_{{\mathrm{i}}} + \beta _{2} {\mathrm{year}}_{{\mathrm{i}}} + {\text{}}\beta _{3} \left( {{\mathrm{educ}}_{{\mathrm{i}}} \times {\mathrm{year}}_{{\mathrm{i}}} } \right) \\ & \quad + {{~}}{\mathbf{\beta }}_{\mathbf{c}} {\mathbf{covariates}}_{{\mathbf{i}}} + \varepsilon _{i} \\ \end{aligned} $$

To address Aim 2, we added the nine factors to Eq. 1. To summarize the percent contribution of each factor, net of all others, to changes in the education-health gradient over the study period, we used the Karlson, Holm, and Breen approach (KHB, 2012). The KHB output summarizes how much each factor attenuated the $\:{\beta\:}_{3}$ interaction coefficient, net of other factors. Although the KHB was developed for nonlinear models, it is directly applicable to linear models.

## Results

Descriptive statistics for Aim 1 are provided in Table 1. The table lists the mean and range for each variable calculated at the state level (*N* = 50), in 1993 and 2019. In 1993, the share of adults in favorable health ranged from 79.2% in the least healthy state to 93.1% in the healthiest state. The average across the 50 states was 88.3%. In 2019, the share of adults in favorable health ranged from 70.7% in the least healthy state to 86.6% in the healthiest state, with an average of 80.8%.

**Table 1:** Summary statistics of study variables across the 50 U.S. States in 1993 and 2019 (*N* = 50)

Variable	1993			2019		
	Average	Min	Max	Average	Min	Max
Favorable self-rated health	0.883	0.792	0.931	0.808	0.707	0.866
Educational attainment (years)	13.859	12.609	14.755	14.642	13.979	15.366
*Individual-level factors*						
Household income (1000$)	43.556	21.503	61.969	67.043	54.743	77.531
Employment						
Currently employed	0.755	0.641	0.838	0.701	0.580	0.795
Unable to work	0.035	0.011	0.088	0.109	0.049	0.215
All others	0.209	0.136	0.314	0.190	0.138	0.243
Has healthcare coverage	0.875	0.785	0.940	0.883	0.760	0.939
Currently married	0.665	0.585	0.764	0.592	0.518	0.743
Current smoker	0.260	0.179	0.339	0.188	0.091	0.271
Obese	0.159	0.107	0.207	0.367	0.268	0.483
*State-level contextual factors*						
Bachelor’s or higher	0.283	0.156	0.404	0.311	0.202	0.431
Immigrants	0.056	0.009	0.230	0.092	0.016	0.267
Policy context score (index)	− 0.052	− 2.185	1.972	0.025	− 3.479	2.912
*Demographics*						
Age						
30–34 years	0.195	0.148	0.237	0.107	0.074	0.148
35–39 years	0.198	0.160	0.231	0.115	0.086	0.155
40–44 years	0.171	0.134	0.199	0.113	0.089	0.155
45–49 years	0.139	0.115	0.175	0.122	0.104	0.138
50–54 years	0.111	0.082	0.147	0.148	0.124	0.172
55–59 years	0.092	0.053	0.119	0.183	0.139	0.215
60–64 years	0.094	0.047	0.125	0.211	0.149	0.271
*Sex*						
Female	0.564	0.518	0.627	0.540	0.497	0.587
Male	0.436	0.373	0.482	0.460	0.413	0.503
*Race*						
Non-Hispanic White	0.839	0.394	0.976	0.747	0.314	0.947
Other	0.161	0.024	0.606	0.253	0.053	0.686

All numbers are proportions unless noted otherwise. From the BRFSS sample of 4,904,293 adults ages 30–64 with information on self-rated health, education, age, sex, and race, we calculated the average of each variable for each state and year. The table shows the average, minimum, and maximum values of those 50 estimates for the first year (1993) and last year (2019) of the 27 years of data used in the analyses. The statistics for income in the table are from 1994 because the income response categories in 1993 were different from all other years

## Aim 1: How did the Gradient Change During 1993 to 2019 in Each U.S. State?

For each state, we estimated Eq. 1. The estimated coefficients for educational attainment, year, and the education-by-year interaction are listed in Table 2 (coefficients for all variables in the models are provided in Table S2 in the Online Supplement). During the 1993–2019 period, the education-health gradient became significantly stronger (*p* < 0.01) in 49 states but changed little for Texas. For example, in Georgia, the education coefficient was 0.019, the year coefficient was − 0.374, and the interaction coefficient was 0.020 (*p* < 0.001). When interpreting the coefficients, recall that the year variable was normalized from 0 to 1 across 1993 to 2019. In Georgia in 1993, each year of education was associated with a 1.9% higher probability of favorable health, net of covariates. In 2019, each year of education was associated with a 3.9% higher probability of favorable health (0.039 = 0.019 + 1*0.020), net of covariates.

**Table 2:** Regression coefficients (and standard errors) predicting the probability of reporting favorable health among adults ages 30–64 years, by U.S. State, 1993–2019

State	Intercept	Education	Year	Education-by-year interaction	*p*-value for interaction
Alabama	0.476	0.030	− 0.222	0.009	< 0.001
	(0.018)	(0.001)	(0.027)	(0.002)	
Alaska	0.736	0.012	− 0.212	0.012	< 0.001
	(0.018)	(0.001)	(0.030)	(0.002)	
Arizona	0.711	0.014	− 0.339	0.018	< 0.001
	(0.017)	(0.001)	(0.025)	(0.002)	
Arkansas	0.487	0.028	− 0.191	0.008	< 0.001
	(0.018)	(0.001)	(0.030)	(0.002)	
California	0.571	0.023	− 0.165	0.008	< 0.001
	(0.012)	(0.001)	(0.017)	(0.001)	
Colorado	0.634	0.017	− 0.152	0.008	< 0.001
	(0.015)	(0.001)	(0.023)	(0.001)	
Connecticut	0.672	0.015	− 0.242	0.014	< 0.001
	(0.015)	(0.001)	(0.023)	(0.001)	
Delaware	0.676	0.016	− 0.236	0.013	< 0.001
	(0.017)	(0.001)	(0.027)	(0.002)	
Florida	0.628	0.021	− 0.313	0.014	< 0.001
	(0.012)	(0.001)	(0.018)	(0.001)	
Georgia	0.670	0.019	− 0.374	0.020	< 0.001
	(0.014)	(0.001)	(0.022)	(0.001)	
Hawaii	0.720	0.014	− 0.146	0.007	< 0.001
	(0.016)	(0.001)	(0.025)	(0.002)	
Idaho	0.655	0.017	− 0.195	0.009	< 0.001
	(0.015)	(0.001)	(0.025)	(0.002)	
Illinois	0.633	0.017	− 0.223	0.012	< 0.001
	(0.015)	(0.001)	(0.025)	(0.002)	
Indiana	0.586	0.021	− 0.238	0.012	< 0.001
	(0.016)	(0.001)	(0.023)	(0.002)	
Iowa	0.665	0.014	− 0.169	0.009	< 0.001
	(0.015)	(0.001)	(0.022)	(0.001)	
Kansas	0.695	0.014	− 0.253	0.014	< 0.001
	(0.015)	(0.001)	(0.022)	(0.001)	
Kentucky	0.348	0.038	− 0.137	0.004	0.004
	(0.013)	(0.001)	(0.020)	(0.001)	
Louisiana	0.606	0.022	− 0.314	0.015	< 0.001
	(0.017)	(0.001)	(0.027)	(0.002)	
Maine	0.586	0.018	− 0.252	0.013	< 0.001
	(0.018)	(0.001)	(0.025)	(0.002)	
Maryland	0.687	0.016	− 0.231	0.013	< 0.001
	(0.012)	(0.001)	(0.018)	(0.001)	
Massachusetts	0.554	0.021	− 0.207	0.011	< 0.001
	(0.014)	(0.001)	(0.022)	(0.001)	
Michigan	0.641	0.016	− 0.309	0.017	< 0.001
	(0.015)	(0.001)	(0.024)	(0.001)	
Minnesota	0.713	0.012	− 0.211	0.011	< 0.001
	(0.013)	(0.001)	(0.018)	(0.001)	
Mississippi	0.448	0.030	− 0.137	0.006	< 0.001
	(0.018)	(0.001)	(0.028)	(0.002)	
Missouri	0.568	0.022	− 0.274	0.013	< 0.001
	(0.017)	(0.001)	(0.027)	(0.002)	
Montana	0.615	0.017	− 0.181	0.009	< 0.001
	(0.019)	(0.001)	(0.029)	(0.002)	
Nebraska	0.634	0.016	− 0.144	0.008	< 0.001
	(0.015)	(0.001)	(0.021)	(0.001)	
Nevada	0.654	0.017	− 0.220	0.011	< 0.001
	(0.019)	(0.001)	(0.031)	(0.002)	
New Hampshire	0.725	0.013	− 0.352	0.020	< 0.001
	(0.018)	(0.001)	(0.027)	(0.002)	
New Jersey	0.619	0.018	− 0.200	0.011	< 0.001
	(0.015)	(0.001)	(0.024)	(0.001)	
New Mexico	0.553	0.025	− 0.118	0.005	0.003
	(0.016)	(0.001)	(0.025)	(0.002)	
New York	0.688	0.015	− 0.234	0.012	< 0.001
	(0.012)	(0.001)	(0.017)	(0.001)	
North Carolina	0.523	0.026	− 0.193	0.009	< 0.001
	(0.014)	(0.001)	(0.023)	(0.001)	
North Dakota	0.623	0.014	− 0.087	0.005	0.003
	(0.020)	(0.001)	(0.029)	(0.002)	
Ohio	0.616	0.018	− 0.325	0.017	< 0.001
	(0.016)	(0.001)	(0.023)	(0.001)	
Oklahoma	0.600	0.021	− 0.285	0.015	< 0.001
	(0.016)	(0.001)	(0.027)	(0.002)	
Oregon	0.594	0.020	− 0.193	0.009	< 0.001
	(0.016)	(0.001)	(0.026)	(0.002)	
Pennsylvania	0.604	0.018	− 0.241	0.012	< 0.001
	(0.013)	(0.001)	(0.022)	(0.001)	
Rhode Island	0.549	0.021	− 0.171	0.009	< 0.001
	(0.017)	(0.001)	(0.027)	(0.002)	
South Carolina	0.613	0.021	− 0.294	0.016	< 0.001
	(0.014)	(0.001)	(0.022)	(0.001)	
South Dakota	0.669	0.011	− 0.167	0.010	< 0.001
	(0.015)	(0.001)	(0.023)	(0.002)	
Tennessee	0.487	0.031	− 0.234	0.010	< 0.001
	(0.016)	(0.001)	(0.025)	(0.002)	
Texas	0.469	0.028	0.001	− 0.002	0.181
	(0.013)	(0.001)	(0.019)	(0.001)	
Utah	0.664	0.014	− 0.148	0.009	< 0.001
	(0.016)	(0.001)	(0.022)	(0.001)	
Vermont	0.609	0.018	− 0.187	0.010	< 0.001
	(0.015)	(0.001)	(0.024)	(0.001)	
Virginia	0.590	0.023	− 0.209	0.010	< 0.001
	(0.015)	(0.001)	(0.023)	(0.001)	
Washington	0.636	0.018	− 0.210	0.011	< 0.001
	(0.012)	(0.001)	(0.019)	(0.001)	
West Virginia	0.389	0.035	− 0.183	0.008	< 0.001
	(0.018)	(0.001)	(0.026)	(0.002)	
Wisconsin	0.672	0.013	− 0.228	0.012	< 0.001
	(0.017)	(0.001)	(0.027)	(0.002)	
Wyoming	0.695	0.013	− 0.227	0.012	< 0.001
	(0.018)	(0.001)	(0.029)	(0.002)	

Sample size = 4,904,293. Coefficients and standard errors are from linear probability models stratified by state. The models also adjust for respondents’ sex, age, race, and sample frame. A table with coefficients and standard errors for all variables is provided in Table S2 in the online supplement

**Fig. 1:**
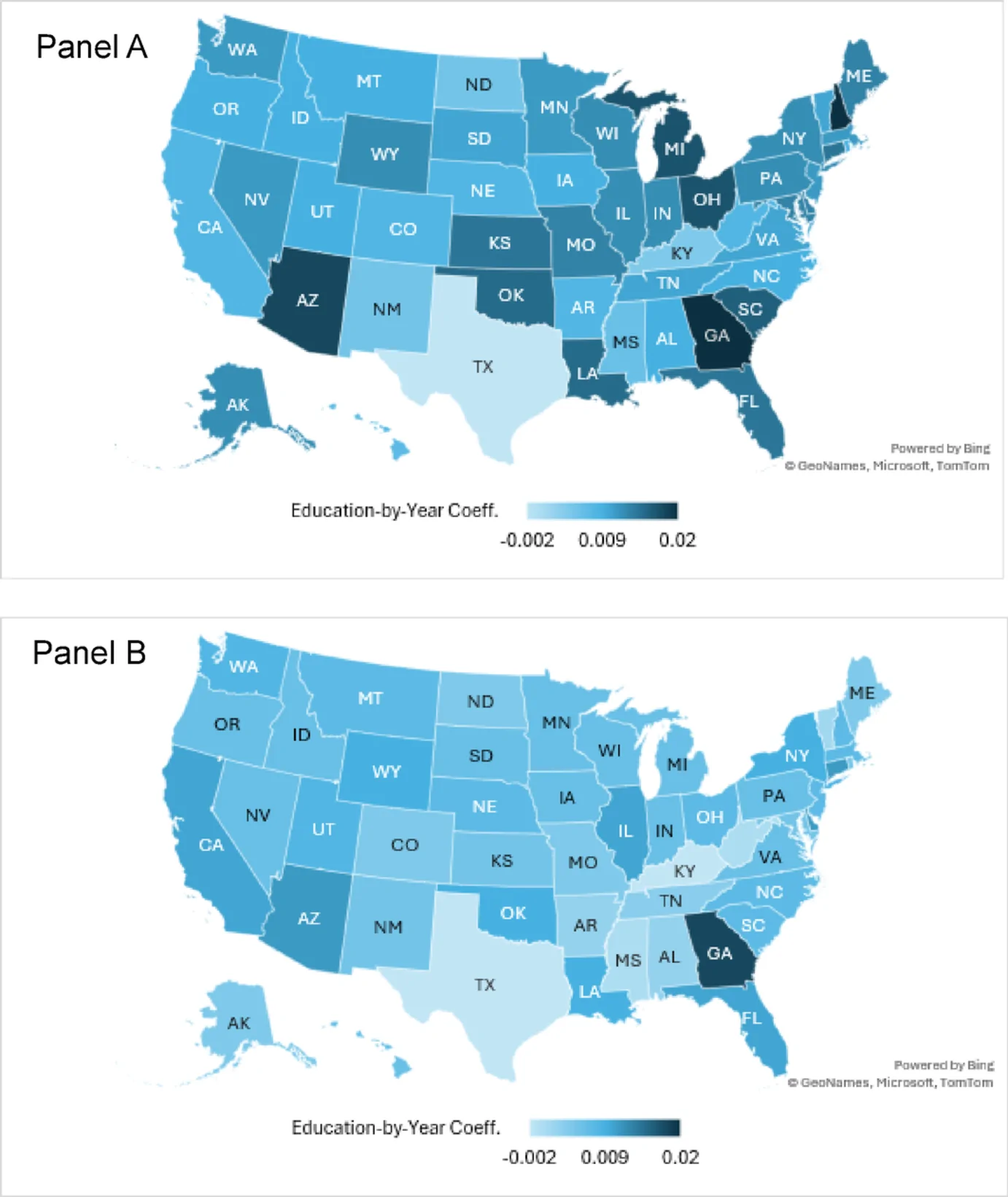
Growth in the education-health gradient in each state. Panel A includes the analytic sample from Aim 1 from 1993 to 2019 including adults of all employment statuses. States in panel A are shaded according to the size of their education-by year coefficients in Table 2. Panel B includes the analytic sample from Aim 2 from 1994 to 2019 which removed adults who were unable to work. States in panel B are shaded according to the size of their education-by year coefficients listed in Table S4 in the Online Supplement

The magnitude of the increase in the education-health gradient differed across states, as shown in Fig. 1, panel A. The states with the largest and smallest increases in the gradient are scattered across the country. The largest increases were in the South (Georgia, South Carolina), Northeast (New Hampshire), West (Arizona), and Midwest (Michigan, Ohio) Census regions. In these states, the increase in favorable health for each year of education was 1.6–2.0 percentage points greater in 2019 than it was in 1993. States with the smallest increases in the gradient also spanned the South (Kentucky, Mississippi, Texas), West (New Mexico, Hawaii), and Midwest (North Dakota) Census regions. In six of these seven states, the interaction coefficients ranged from 0.004 to 0.007 (recall, the coefficient for Texas was − 0.002). In these states, the increase in favorable health for each year of education was 0.4–0.7 percentage points greater in 2019 than it was in 1993, net of covariates. In Online Supplement Fig. S1, we provide a figure for each state, showing how the probability of favorable health changed over time for adults with a high school credential and those with a bachelor’s degree or higher.

## Aim 2: How Much of the Change in the Gradient in Each State Can be Statistically Accounted for by the Nine Hypothesized Factors?

Recall that, for this aim, we excluded respondents who stated that they were unable to work. Before we estimated the contribution of the nine factors to the increasing education-health gradient among this sample, we estimated how the gradient changed over time for this sample in each state, again using Eq. 1. The results are displayed in panel B of Fig. 1 (descriptive statistics and all model coefficients are in Online Supplement Tables S3 and S4, respectively). Most states have a lighter shade in panel B than panel A, indicating that the gradient increased less among adults who were able to work. Among this sample of adults, there was no increase in the gradient for nine states using a conventional *α* = 0.05 threshold for statistical significance: Alabama, Arkansas, Kentucky, Mississippi, Missouri, Tennessee, Vermont, and West Virginia, plus Texas where the gradient did not change even among all adults as whole.

For the 41 states with a statistically significant increase in the gradient, we estimated how much of the increase was accounted for by the nine factors. These estimates, produced by the KHB output, are in Table 3. The table presents results by state, starting with Maine, the state with the largest proportion of increase accounted for by all factors together. The factors accounted for a sizable amount of the increase in many states but a small amount in others. For example, they accounted for all of the increase in Maine, 90.9% of the increase in Alaska, and 66.7% of the increase in South Dakota versus just 10.3% in Utah, 10.4% in Connecticut, and 5.6% in Delaware.

**Table 3:** Contribution of nine factors to the increase in the gradient among adults ages 30–64 who were able to work, by U.S. State, 1994–2019

State	Total	Six Individual-level factors						Three state-level contextual factors			
		Income	Employment	Healthcare	Marriage	Obesity	Smoking	College graduates	Immigrants	Policy contexts	*p*-value for interaction in full model^a^
Maine	1.026	0.376	0.152	0.001	0.031	0.162	0.099	0.113	0.054	0.038	0.946
Alaska	0.909	0.436	0.100	− 0.003	0.091	0.153	0.075	0.022	0.016	0.019	< 0.001
South Dakota	0.667	0.342	0.081	0.030	0.044	0.059	0.080	0.073	− 0.045	0.003	0.218
South Carolina	0.648	0.353	0.113	0.062	0.007	0.020	0.053	0.009	− 0.030	0.061	0.103
Montana	0.613	0.345	0.054	− 0.003	0.070	0.045	0.042	0.013	0.046	0.002	0.180
New Mexico	0.556	0.521	0.064	− 0.051	− 0.018	0.039	− 0.044	0.007	0.029	0.008	0.252
Oregon	0.538	0.204	0.067	0.005	0.051	0.207	− 0.051	0.003	0.057	− 0.005	0.134
Michigan	0.533	0.266	0.103	0.010	0.056	0.047	0.031	0.023	− 0.015	0.011	0.050
Oklahoma	0.530	0.312	0.106	0.011	0.001	0.023	0.017	0.013	0.046	0.001	0.020
Arizona	0.499	0.292	0.026	0.001	0.013	0.087	0.023	0.027	0.016	0.014	0.003
North Dakota	0.495	0.178	− 0.038	− 0.013	0.076	0.144	0.081	0.043	0.035	− 0.012	0.262
Rhode Island	0.493	0.130	0.122	0.032	− 0.001	0.119	− 0.019	0.082	0.098	− 0.071	0.198
Ohio	0.491	0.269	0.077	0.013	0.027	0.031	0.051	0.017	0.007	0.000	0.017
Colorado	0.432	0.102	0.088	0.035	0.026	0.092	− 0.054	0.052	0.049	0.042	0.134
North Carolina	0.431	0.201	0.094	0.166	− 0.008	0.020	− 0.032	0.020	− 0.005	− 0.026	0.020
Louisiana	0.419	0.215	0.065	− 0.040	0.002	0.040	0.053	0.012	0.061	0.011	0.011
Kansas	0.415	0.163	0.101	0.059	0.026	0.065	0.010	0.057	0.021	− 0.087	0.040
Indiana	0.400	0.221	0.123	0.009	0.014	0.043	− 0.035	0.027	− 0.026	0.023	0.023
New Hampshire	0.397	0.121	0.072	0.006	0.027	0.094	0.014	0.040	− 0.012	0.037	0.008
Pennsylvania	0.393	0.103	0.085	− 0.003	0.037	0.089	0.037	0.029	− 0.001	0.017	0.010
Georgia	0.389	0.199	0.063	0.044	0.002	0.020	0.026	0.013	− 0.020	0.040	< 0.001
New York	0.332	0.153	0.044	0.002	0.022	0.064	0.016	− 0.015	0.049	− 0.004	< 0.001
Massachusetts	0.325	0.103	0.119	− 0.009	0.023	0.068	− 0.015	0.041	0.028	− 0.033	0.001
Hawaii	0.325	0.124	0.021	− 0.012	0.036	0.175	− 0.010	0.034	− 0.004	− 0.039	0.009
Virginia	0.310	0.074	0.078	0.056	0.021	0.064	− 0.001	− 0.024	0.037	0.004	0.012
Maryland	0.288	0.069	0.077	− 0.006	0.013	0.066	− 0.033	0.051	0.025	0.025	0.002
Wisconsin	0.288	0.131	0.008	0.011	0.054	0.047	− 0.012	0.035	0.059	− 0.045	0.016
Florida	0.285	0.167	0.057	0.014	0.015	− 0.030	0.028	0.007	− 0.003	0.031	< 0.001
Idaho	0.262	0.145	0.040	0.032	0.010	0.034	− 0.055	0.025	0.030	0.001	0.007
Illinois	0.261	0.123	0.033	0.064	0.009	0.025	− 0.026	0.006	0.036	− 0.008	< 0.001
Wyoming	0.257	0.099	0.036	0.012	0.022	0.042	− 0.011	0.026	0.033	− 0.001	< 0.001
Iowa	0.249	0.131	0.015	0.002	0.029	0.074	− 0.040	0.030	0.010	− 0.002	0.002
New Jersey	0.232	0.006	0.074	0.026	0.009	0.094	− 0.014	0.032	− 0.001	0.007	0.001
Minnesota	0.192	0.025	0.017	0.009	0.049	0.087	0.003	0.029	− 0.015	− 0.012	< 0.001
Washington	0.169	0.081	0.025	0.008	0.023	0.098	− 0.090	0.010	0.016	− 0.002	< 0.001
California	0.151	0.102	0.008	− 0.001	0.004	0.040	− 0.014	0.003	0.006	0.002	< 0.001
Nebraska	0.148	0.069	0.034	0.050	0.022	− 0.013	− 0.052	0.016	0.026	− 0.004	< 0.001
Nevada	0.134	0.181	0.100	0.028	− 0.005	0.035	− 0.251	0.022	0.012	0.012	0.022
Connecticut	0.104	0.013	0.056	0.007	− 0.001	0.042	− 0.035	0.025	− 0.004	0.002	< 0.001
Utah	0.103	0.007	0.028	0.011	0.030	0.061	− 0.055	0.006	0.018	− 0.003	< 0.001
Delaware	0.056	0.055	0.064	0.003	0.002	− 0.029	− 0.054	0.031	− 0.012	− 0.005	< 0.001
Average	0.384	0.176	0.065	0.017	0.023	0.064	− 0.006	0.026	0.018	0.001	
Minimum	0.056	0.006	− 0.038	− 0.051	− 0.018	− 0.030	− 0.251	− 0.024	− 0.045	− 0.087	
Maximum	1.026	0.521	0.152	0.166	0.091	0.207	0.099	0.113	0.098	0.061	
Contribution > 0	41	41	40	31	36	38	18	39	27	23	
Contribution < 0	0	0	1	10	5	3	23	2	14	17	

Models are stratified by state. All models account for sex, age, race, and sample frame

^a^ The* p*-values are for the education-by-year interaction term in the full regression model with all nine factors and covariates. Small p-values, such as those less than 0.05, indicate that the factors did not fully account for the increasing education-health gradient during the study period

Table 3 also shows whether the increase in the gradient remained statistically significant once all nine factors were in the model. Specifically, the last column lists the *p*-values for each state’s education-by-year interaction coefficients when all factors are in the model. They are larger than 0.05 for nine states and larger than 0.01 for 19 states. In sum, taken together, the nine factors were better explanations for the increasing gradient in some states than others.

**Fig. 2:**
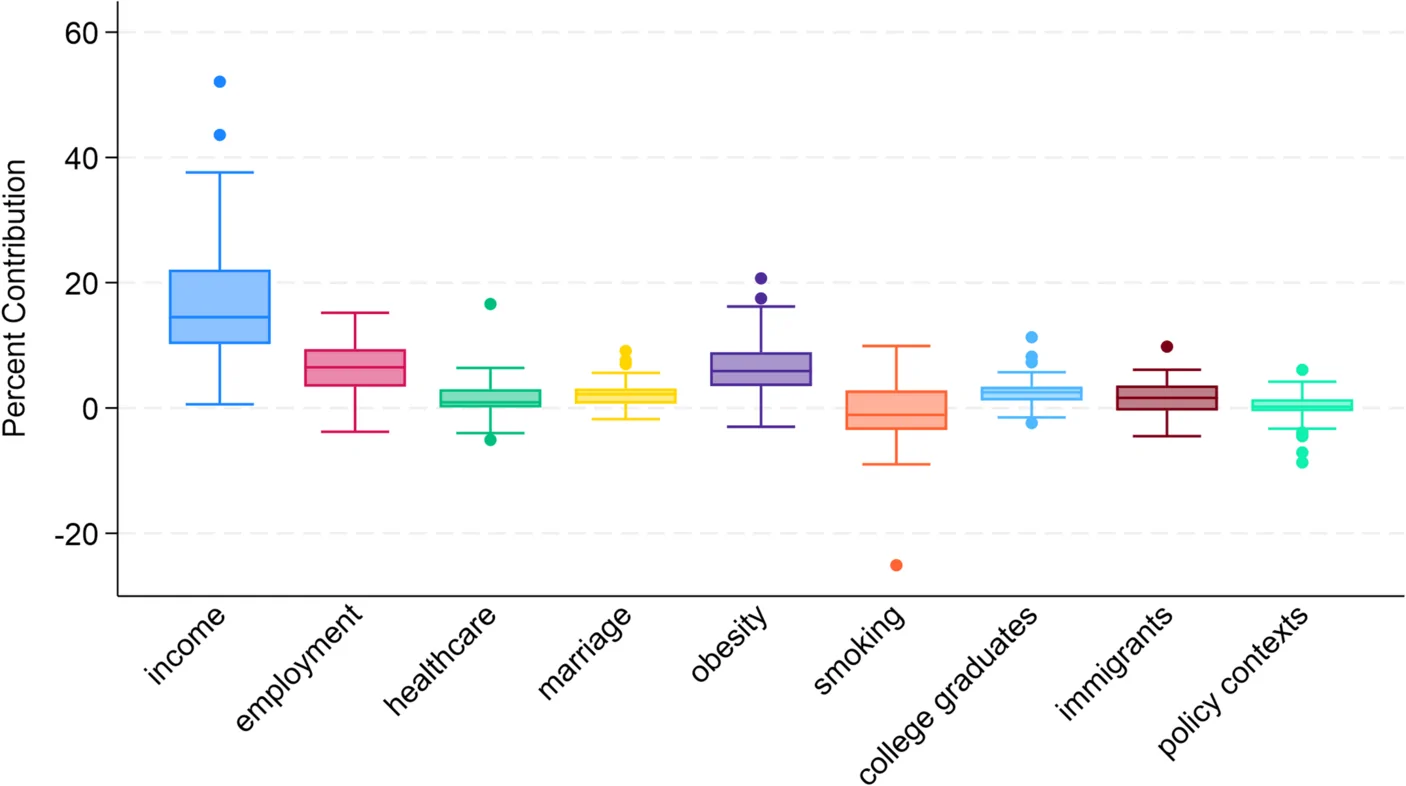
Contribution of each factor to the increase in the gradient from 1994 to 2019, by U.S. State (*N* = 41). Analyses include adults ages 30–64 who reported that they were able to work. The top and bottom of each box represent the first and third quartiles of the data, respectively, while the middle line represents the median. The lines, often called “whiskers,” extend to 1.5 times the interquartile range. The data used to create the box plots are taken directly from Table 3. The five key values shown in the boxplots (first, second, and third quartile, and the upper and lower whiskers) are provided in Table S5 in the Online Supplement

### How Much did Specific Factors Contribute to the Increasing Gradient?

Table 3 also lists the contribution of each of the nine factors to changes in the gradient in each state. We summarized the distribution of the contribution of each factor with box plots in Fig. 2. The figure shows that income was a large contributor in most states, net of the other eight factors. Still, the size of its contribution notably differed across states. The median contribution of income to the increasing gradient across the 41 states was 14.5%, net of other factors. That is, in about half of these states, 14.5% or more of the increase appears to reflect a strengthening link between education, income, and health. The first and third quartiles of income’s contribution across the states were 10.2% and 22.1%, respectively. The minimum was 0.7% in Utah and New Jersey and the maximum was 52.1% in New Mexico.

The contribution of two other factors, employment and obesity, also stood out in Fig. 2, albeit less so than that of income. The median contribution of employment to changes in the gradient among the 41 states was 6.5%. The first and third quartiles were 3.4% and 9.4%, respectively. In other words, in about half of the analyzed states, 6.5% or more of the increase in the gradient appeared to be due to a strengthening link between education, employment, and health. The contribution of employment was negative in North Dakota, suggesting that the link between education, employment, and health weakened in that state. The median contribution of obesity to changes in the gradient was 5.9%, with first and third quartiles of 3.5% and 8.9%, respectively. The contribution was small and negative in Delaware, Florida, and Nebraska.

Unlike any other factor, the contribution of smoking to changes in the education-health association was negative in most states (23 out of 41). In these states, changes in the link between education, smoking, and health appear to have suppressed increases in the gradient to a small degree. The median contribution of smoking to changes in the gradient was − 1.1%, with first and third quartiles of -3.5% and 2.8%, respectively. The outlier for smoking is Nevada, where the contribution was − 25.1%. The contributions of healthcare coverage and marital status were small, as were the contributions of the three-state level factors. Table S5 in the Online Supplement lists the values shown in each box plot for each of the nine factors.

### How Well do the Findings Hold Within Specific States?

**Fig. 3:**
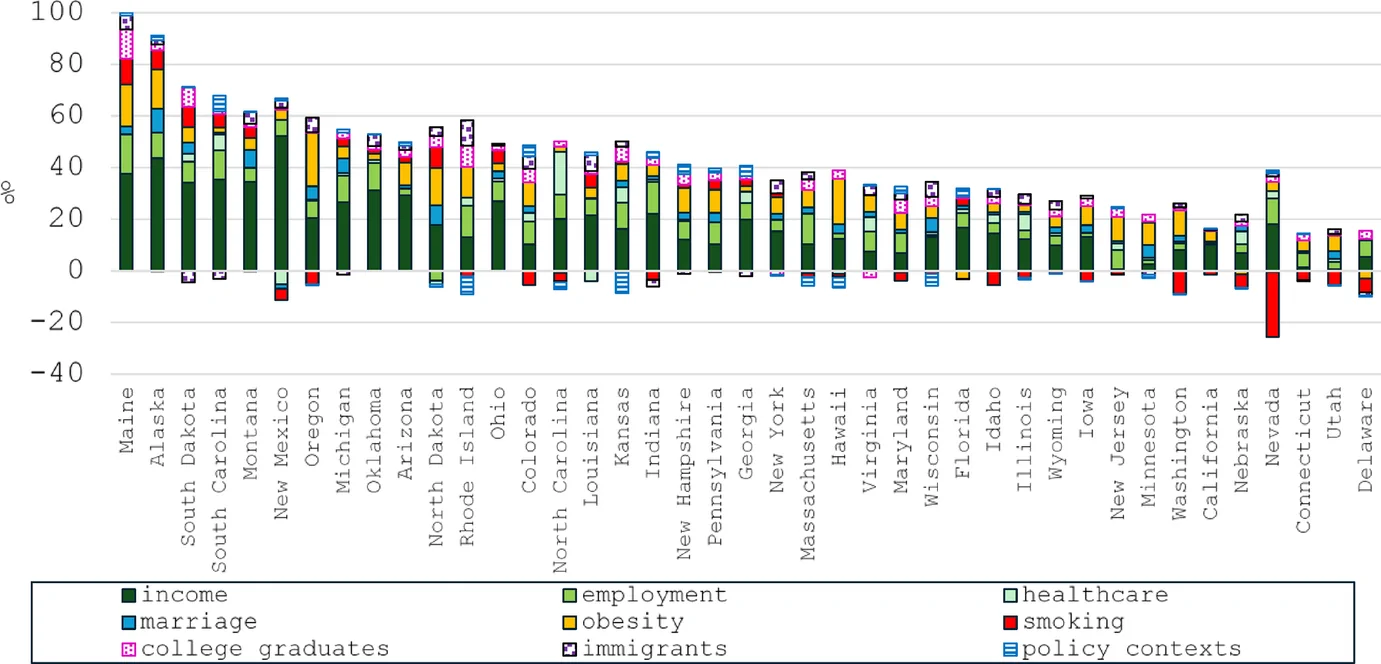
Contribution of each factor to the increase in the gradient from 1994 to 2019, by U.S. State (*N* = 41). Bars are ordered from left to right by the total contribution of all factors to the increasing gradient, from largest to smallest. The underlying data are from Table 3

While Fig. 2 summarizes the results across the 41 states, Fig. 3 shows results separately for each state. Each bar contains the percentage contribution of the nine factors to the increasing gradient. We note two key patterns. First, again, income tended to be the largest contributor to increasing gradient across each state. Among the 41 states, the contribution of income to the increasing gradient was greater than that of employment in 35 states, greater than that of obesity in 34 states, and greater than that of smoking in all states. Second, in 37 of the 41 states, the combined contribution of two economic factors (income and employment) to the increasing gradient exceeded the combined contribution of smoking and obesity. The exceptions are Minnesota, Hawaii, and North Dakota, where the combined contribution of the economic factors was smaller than that for the behavioral factors, plus New Jersey, where the combined contributions were similar. Lastly, the Figure is useful for identifying where state-level contextual factors contributed to changes in the gradient, albeit marginally.

### Ancillary Analyses

As explained earlier, the analyses in Aim 2 excluded adults who were unable to work because this employment category is effectively a proxy for adverse health. Nevertheless, to address potential concerns that it is not a perfect proxy and that there may be differences across states in how much of an obstacle adverse health is to employment, we replicated the Aim 2 analyses with all adults. To do so, we modified the binary employment measure by combining adults unable to work with all other not-employed adults (results are shown in Online Supplement Fig. S2). As expected, given our endogeneity concerns, the contribution of employment to the increasing gradient was larger in these analyses and rivaled the contribution of income. This was especially evident in the nine states whose increase in the gradient in the main analyses was not significant when adults who were unable to work were excluded. Nevertheless, the core conclusions are consistent between the two analyses: the main explanation for the increasing gradient across most states is changes in economic conditions, measured by income and employment.

In other ancillary analyses, we re-estimated the models using a binary logistic regression to confirm that the estimated probabilities were similar to those obtained from the linear probability model. In addition, to ensure that excluding respondents with missing data on any of the six individual-level factors did not impact our study’s conclusion, we conducted an auxiliary imputation. Because the KHB does not provide variable-specific decompositions for a multiply-imputed dataset, we chose the following approach. We imputed missing values for our analyses in Aim 2 using multivariate chained equations. We then extracted at random one imputed dataset and replicated the KHB mediation analysis. Our study’s conclusions are unchanged. Lastly, we assessed whether the small contribution of the state policy factor might be due to our inclusion of myriad pathways through which state policies can shape health. For example, state policies on minimum wage and tobacco taxes can affect individual-level income and smoking. We re-estimated the KHB with only state policy, but its contribution remained small.

### Discussion and Conclusion

The association between educational attainment level and health among U.S. adults has become stronger in recent decades, but questions remain about why the trend has occurred. Our study advances this line of research by investigating geographic heterogeneity in how and why the education-health gradient has changed in recent decades. Using data on adults ages 30–64 over a multi-decade period, we documented how the association between educational attainment and self-rated health changed within each U.S. state and estimated the contribution of nine factors to the changes. Next, we discuss six key findings.

The first key finding is that the association between educational attainment level and self-rated health grew stronger during the study period in all states except Texas; however, the magnitude of the growth differed across states. Our finding contrasts with a prior study of mortality, not self-rated health, which reported that the education-mortality gradient among adults ages 45–89 increased in some states but not in others during an earlier, 1985–2011, time period (Montez et al. 2019b). In states with the largest growth in the gradient, the increase in the probability of reporting favorable self-rated health occurring with each additional year of education was 2 percentage points greater in 2019 than it was in 1993. In states with the smallest growth in the gradient, the increase in the probability of reporting favorable self-rated health with each additional year of education was 0.4 percentage points greater in 2019 than it was in 1993. These seemingly small percentage point increases add up to large differences over time. For example, in Georgia, a state with a large increase in the gradient, adults with a bachelor’s degree had an 8% higher probability of favorable health than their peers with a high school credential in 1993 but a 16% higher probability in 2019.

Growth in the education-health gradient was attenuated after we removed adults from the sample who indicated that they were unable to work, to address concerns about endogeneity between health and ability to work. After removing these adults, growth in the gradient across nine additional states was no longer statistically significant. In these states, the relationship between education, ability to work, and health appears to be particularly tightly interconnected. Interestingly, eight of the nine states share a fairly conservative orientation toward economic safety nets, labor laws, and Medicaid. It could be the case that, in those states in particular, adults with lower educational attainment are more likely to develop health problems which prevent them from employment due to myriad factors such as lack of health care or employer accommodations. In this case, being unable to work is effectively *a proxy* for unfavorable health. Alternatively, in those states, people with early-life disabilities may experience especially high obstacles to education and employment opportunities, which subsequently damages health. In this case, being unable to work is a risk factor for unfavorable health. The cross-sectional data do not allow us to adjudicate the temporal order of education, ability to work, and health. The remainder of findings discussed below apply to the 92% of the sample who stated they were able to work. These adults tended to be advantaged in other factors such as income, marital status, smoking, and obesity. Nevertheless, for both adults who were able to work and those unable to work, income and employment emerged as the key contributors to the increasing gradient.

A second key finding is that, across most states, household income contributed more to the increasing gradient than any other factor that we examined. The size of income’s contribution was also the most varied across states, ranging from just 0.7% in Utah and New Jersey to a striking 52.1% in New Mexico. The comparatively large contribution of income in most states concurs with a study of U.S. adults which concluded that the association between educational attainment and self-rated health has become stronger across birth cohorts in large part because the pathways from education to income to health have become stronger (Lynch, 2006). We found that the importance of income for the growing gradient was striking overall, yet its importance was neither universal nor inevitable. Understanding why income had a larger role in some states than others is a question for future research.

Our third key finding is that, across most states, the increasing gradient was more attributable to income and employment than to smoking and obesity. In other words, the story is largely an economic one. An economic story is consistent with the narrative put forward by others regarding the destruction of good jobs for adults without a bachelor’s degree, shuttering of factories, declining social mobility, and similar adverse trends that have far-reaching, negative effects on the health and wellbeing of adults with lower educational attainment (Case & Deaton, 2020; Venkataramani et al., 2020). One implication is that efforts to further flesh out explanations for the strengthening gradient as well as actions to reduce it might be most effective by targeting the economic dimensions. The contrasts across states in this study provide ample purchase for doing so. For instance, what happened in states like New Jersey, Utah, Connecticut, and Minnesota where income contributed less than 3% to the increasing gradient, compared to states like New Mexico, Alaska, Maine, and South Carolina where it contributed over 35%?

Fourth, unlike any other factor, smoking decreased the gradient in most states. Specifically, in 23 of the 41 states in which we examined the contribution of the nine factors, changes occurring along the pathways between education, smoking, and health appeared to have operated in a manner that weakened the gradient. The finding echoes studies of education-mortality association at the national level in recent decades, where the educational gradient in smoking-related causes of death has recently weakened for men (Ho & Fenelon, 2015; Sasson & Hayward, 2019). This is a departure from evidence from the latter decades of the twentieth century where smoking was a primary explanation for the increasing education-mortality gradient, as adults with higher educational attainment quit or avoided smoking following the 1964 Surgeon General’s report (Link, 2008; Meara et al., 2008).

Our fifth key finding is that, even though some factors appear to have weakened the gradient during the study period, in no state were those changes large enough to overcome the contribution of factors that strengthened the gradient. This finding comports with a core argument of FCT. That is, even though the mechanisms linking education to health tend to change over time, the gradient nevertheless persists and can even grow, as the salience of some mechanisms are replaced by others (Link & Phelan, 1995; Phelan et al., 2010).

Lastly, there was no single explanation for the increasing gradient across all states. This too is aligned with FCT, which posits that the education-health association persists in part because of the numerous and varied pathways between educational attainment and health. One implication of our finding is that a national effort combined with state-specific efforts might be necessary. If there were to be a national effort, bolstering the incomes of persons with lower educational attainment through, for example, raising the federal minimum wage or providing robust employment opportunities, may be among strategies to consider. Although no single explanation emerged, our overall findings indicate that broader economic and labor conditions, rather than adults’ health behaviors or the changing composition of adults who attain certain education levels, were the main contributors to the increasing gradient across most states.

### Limitations and Future Directions

The BRFSS is the best dataset for our study because it is representative within each state, has a large sample size, and spans multiple decades, but it has limitations. First, the BRFSS data are cross-sectional, so we cannot assess causality or determine the temporal order between educational attainment, the individual-level factors, and health. Thus, we cannot claim that our estimates reflect causal effects from education to the mediators to adult health, as the estimates may also contain an effect of early-life health, or other early-life circumstances, on educational attainment. That said, multiple studies using longitudinal data and causal inference methods to assess the causal order between educational attainment and self-rated health conclude that it runs from the former to the latter (Luo & Wei, 2024; Warren, 2009). Second, while our six individual-level factors capture the main domains widely studied in this literature, the particular indicators we used to measure them were limited to those available in the BRFSS. Additional measures may help explain more of the increasing gradient. This critique is particularly relevant for states where the factors explained a relatively small portion of the increasing gradient.

We also note that the complexity of the analysis (e.g., estimating and interpreting education-by-time interactions and nine potential explanatory factors for those interactions across 50 states) and, in particular, our objective to compare changes in the gradient across states required simplifying assumptions such as a linear association between education and health and a linear time trend across all 50 states. Alternatives include using a more complex functional form for the education-health association (i.e., step-changes) and year (i.e., quadratic) on all 50 states, or using functional forms that are optimized specifically for each state. In both cases, the costs to interpretability and to the ability to compare the gradient across states far exceed any benefits from the added complexity. Using a linear functional form for all states, our estimates may be somewhat less precise than if we had used functional forms specific to each state but the consistency in our findings across states in Aim 2 provides confidence in the conclusions. We also note that in ancillary analyses, we estimated changes in the gradient using a binary logistic regression model to allow for a nonlinear relationship between educational attainment and the probability of favorable health, and found similar estimates of how the gradient changed in each state. Lastly, exploring potential variation across demographic subgroups, such as race or age, and the factors underlying the variation, is important but beyond the scope of the present study.

## Conclusions

The association between adults’ educational attainment and their self-reported health grew significantly stronger in 49 of the 50 U.S. states during the 1993–2019 period. Despite the ubiquity of the growth, its magnitude notably differed across states. The differences support the notion that the growth is shaped by contexts as posited in FCT. Across most states, the largest contributor to the growing gradient was household income. That is, changes over time in the strength of the pathway from education to income to health emerged as a prominent, but not only, explanation for the strengthening gradient. Although no single explanation emerged across all states, our findings indicate that broader economic and labor conditions, rather than health behaviors or the changing composition of adults who attain certain education levels, were the main contributors to the increasing gradient across most states.

## Supplementary Information

Below is the link to the electronic supplementary material.

Supplementary Material 1

## Data Availability

The data are publicly available at https://www.cdc.gov/brfss/index.html.
