# Supplementary material for: Changes in the Education-Health Gradient Within U.S. States, 1993–2019
Source: Popul Res Policy Rev. 2026 Jun 22;45(4):37. doi: 10.1007/s11113-026-10020-8 (PMC13287250; doi:10.1007/s11113-026-10020-8)
Supplement: Supplementary file 1 — Supplementary Material 1 [file 11113_2026_10020_MOESM1_ESM.docx]

**ONLINE SUPPLEMENT**

**Changes in the Education-Health Gradient within U.S. States, 1993-2019**

| **Table S1**  *Summary of Study Variables among Analytic Sample of Adults by Employment Status* | | | | |
| --- | --- | --- | --- | --- |
| Variable |  | Employed | Unable To Work | All Others |
| Favorable self-rated health |  | .910 | .301 | .801 |
| Educational attainment (years) |  | 14.870 | 12.422 | 14.040 |
|  |  |  |  |  |
| Individual-level factors |  |  |  |  |
| Household income (1000$) |  | 67.706 | 26.449 | 52.180 |
| Has healthcare coverage |  | .890 | .884 | .802 |
| Currently married |  | .647 | .346 | .652 |
| Current smoker |  | .188 | .385 | .217 |
| Obese |  | .277 | .454 | .296 |
|  |  |  |  |  |
| Demographics |  |  |  |  |
| Age |  |  |  |  |
| 30-34 years |  | .120 | .039 | .105 |
| 35-39 years |  | .138 | .058 | .103 |
| 40-44 years |  | .149 | .087 | .095 |
| 45-49 years |  | .160 | .133 | .094 |
| 50-54 years |  | .168 | .199 | .117 |
| 55-59 years |  | .154 | .250 | .171 |
| 60-64 years |  | .110 | .236 | .315 |
| Sex |  |  |  |  |
| Female |  | .543 | .617 | .713 |
| Male |  | .457 | .383 | .287 |
| Race |  |  |  |  |
| Non-Hispanic White |  | .804 | .702 | .768 |
| Other |  | .196 | .298 | .232 |

*Note.* All numbers are proportions unless stated otherwise. The columns represent the average among respondents for each variable by employment status across all 27 years of the 1993 to 2019 study period. The BRFSS sample used for the estimates contains 4,904,293 adults ages 30–64 with complete information on self-rated health, education, age, sex, and race.

| **Table S2**  *Regression Coefficients (and Standard Errors) Predicting the Probability of Reporting Favorable Health among Adults Ages 30–64 Years, by U.S. State, 1993–2019* | | | | | | | | | | | | | |
| --- | --- | --- | --- | --- | --- | --- | --- | --- | --- | --- | --- | --- | --- |
| State | Intercept | Education | Year | Education-by-year interaction | Age  35-39 | Age  40-44 | Age  45-49 | Age  50-54 | Age  55-59 | Age  60-64 | Female | Non-Hispanic white | Cellphone Survey Mode |
| Alabama | 0.476 | 0.030 | -0.222 | 0.009 | -0.025 | -0.071 | -0.102 | -0.145 | -0.188 | -0.187 | -0.017 | 0.068 | 0.014 |
|  | (0.018) | (0.001) | (0.027) | (0.002) | (0.005) | (0.005) | (0.005) | (0.005) | (0.005) | (0.005) | (0.003) | (0.003) | (0.004) |
| Alaska | 0.736 | 0.012 | -0.212 | 0.012 | -0.020 | -0.033 | -0.058 | -0.077 | -0.103 | -0.122 | -0.014 | 0.059 | -0.012 |
|  | (0.018) | (0.001) | (0.030) | (0.002) | (0.005) | (0.005) | (0.005) | (0.005) | (0.006) | (0.006) | (0.003) | (0.004) | (0.005) |
| Arizona | 0.711 | 0.014 | -0.339 | 0.018 | -0.014 | -0.049 | -0.072 | -0.108 | -0.133 | -0.138 | -0.012 | 0.061 | 0.004 |
|  | (0.017) | (0.001) | (0.025) | (0.002) | (0.005) | (0.005) | (0.005) | (0.005) | (0.005) | (0.005) | (0.003) | (0.003) | (0.004) |
| Arkansas | 0.487 | 0.028 | -0.191 | 0.008 | -0.026 | -0.063 | -0.104 | -0.142 | -0.177 | -0.185 | -0.012 | 0.075 | -0.014 |
|  | (0.018) | (0.001) | (0.030) | (0.002) | (0.005) | (0.006) | (0.006) | (0.006) | (0.006) | (0.006) | (0.003) | (0.004) | (0.006) |
| California | 0.571 | 0.023 | -0.165 | 0.008 | -0.016 | -0.041 | -0.072 | -0.098 | -0.126 | -0.139 | -0.017 | 0.074 | -0.001 |
|  | (0.012) | (0.001) | (0.017) | (0.001) | (0.003) | (0.003) | (0.003) | (0.003) | (0.004) | (0.004) | (0.002) | (0.002) | (0.003) |
| Colorado | 0.634 | 0.017 | -0.152 | 0.008 | -0.019 | -0.028 | -0.052 | -0.066 | -0.090 | -0.098 | -0.012 | 0.087 | -0.013 |
|  | (0.015) | (0.001) | (0.023) | (0.001) | (0.003) | (0.003) | (0.003) | (0.003) | (0.003) | (0.004) | (0.002) | (0.003) | (0.003) |
| Connecticut | 0.672 | 0.015 | -0.242 | 0.014 | -0.014 | -0.034 | -0.049 | -0.070 | -0.088 | -0.101 | -0.013 | 0.084 | -0.018 |
|  | (0.015) | (0.001) | (0.023) | (0.001) | (0.004) | (0.004) | (0.004) | (0.004) | (0.004) | (0.004) | (0.002) | (0.003) | (0.003) |
| Delaware | 0.676 | 0.016 | -0.236 | 0.013 | -0.006 | -0.032 | -0.067 | -0.084 | -0.108 | -0.117 | -0.016 | 0.056 | -0.018 |
|  | (0.017) | (0.001) | (0.027) | (0.002) | (0.005) | (0.005) | (0.005) | (0.005) | (0.005) | (0.005) | (0.003) | (0.004) | (0.005) |
| Florida | 0.628 | 0.021 | -0.313 | 0.014 | -0.020 | -0.045 | -0.076 | -0.112 | -0.140 | -0.144 | -0.010 | 0.047 | 0.009 |
|  | (0.012) | (0.001) | (0.018) | (0.001) | (0.003) | (0.003) | (0.003) | (0.003) | (0.003) | (0.003) | (0.002) | (0.002) | (0.003) |
| Georgia | 0.670 | 0.019 | -0.374 | 0.020 | -0.018 | -0.042 | -0.079 | -0.113 | -0.150 | -0.159 | -0.017 | 0.040 | 0.007 |
|  | (0.014) | (0.001) | (0.022) | (0.001) | (0.004) | (0.004) | (0.004) | (0.004) | (0.005) | (0.005) | (0.003) | (0.003) | (0.004) |
| Hawaii | 0.720 | 0.014 | -0.146 | 0.007 | -0.014 | -0.032 | -0.050 | -0.080 | -0.096 | -0.100 | -0.003 | 0.055 | -0.008 |
|  | (0.016) | (0.001) | (0.025) | (0.002) | (0.004) | (0.004) | (0.004) | (0.004) | (0.004) | (0.004) | (0.002) | (0.002) | (0.004) |
| Idaho | 0.655 | 0.017 | -0.195 | 0.009 | -0.019 | -0.034 | -0.061 | -0.085 | -0.104 | -0.120 | -0.007 | 0.074 | 0.019 |
|  | (0.015) | (0.001) | (0.025) | (0.002) | (0.004) | (0.004) | (0.004) | (0.004) | (0.005) | (0.005) | (0.002) | (0.005) | (0.004) |
| Illinois | 0.633 | 0.017 | -0.223 | 0.012 | -0.010 | -0.025 | -0.052 | -0.074 | -0.107 | -0.115 | -0.010 | 0.089 | -0.013 |
|  | (0.015) | (0.001) | (0.025) | (0.002) | (0.004) | (0.004) | (0.004) | (0.004) | (0.005) | (0.005) | (0.003) | (0.003) | (0.004) |
| Indiana | 0.586 | 0.021 | -0.238 | 0.012 | -0.017 | -0.042 | -0.070 | -0.093 | -0.121 | -0.125 | -0.016 | 0.077 | -0.017 |
|  | (0.016) | (0.001) | (0.023) | (0.002) | (0.004) | (0.004) | (0.004) | (0.004) | (0.004) | (0.005) | (0.002) | (0.004) | (0.004) |
| Iowa | 0.665 | 0.014 | -0.169 | 0.009 | -0.004 | -0.029 | -0.046 | -0.064 | -0.081 | -0.100 | -0.005 | 0.092 | -0.015 |
|  | (0.015) | (0.001) | (0.022) | (0.001) | (0.004) | (0.004) | (0.004) | (0.004) | (0.004) | (0.004) | (0.002) | (0.006) | (0.003) |
| Kansas | 0.695 | 0.014 | -0.253 | 0.014 | -0.013 | -0.034 | -0.057 | -0.079 | -0.099 | -0.114 | -0.013 | 0.074 | -0.022 |
|  | (0.015) | (0.001) | (0.022) | (0.001) | (0.003) | (0.003) | (0.003) | (0.003) | (0.003) | (0.003) | (0.002) | (0.003) | (0.003) |
| Kentucky | 0.348 | 0.038 | -0.137 | 0.004 | -0.017 | -0.054 | -0.098 | -0.130 | -0.169 | -0.174 | -0.012 | 0.038 | 0.022 |
|  | (0.013) | (0.001) | (0.020) | (0.001) | (0.004) | (0.004) | (0.004) | (0.004) | (0.005) | (0.005) | (0.003) | (0.004) | (0.004) |
| Louisiana | 0.606 | 0.022 | -0.314 | 0.015 | -0.024 | -0.054 | -0.086 | -0.129 | -0.169 | -0.184 | -0.011 | 0.070 | 0.016 |
|  | (0.017) | (0.001) | (0.027) | (0.002) | (0.004) | (0.005) | (0.005) | (0.005) | (0.005) | (0.005) | (0.003) | (0.003) | (0.005) |
| Maine | 0.586 | 0.018 | -0.252 | 0.013 | -0.011 | -0.028 | -0.048 | -0.071 | -0.089 | -0.095 | -0.003 | 0.099 | -0.012 |
|  | (0.018) | (0.001) | (0.025) | (0.002) | (0.004) | (0.004) | (0.004) | (0.004) | (0.004) | (0.004) | (0.002) | (0.007) | (0.004) |
| Maryland | 0.687 | 0.016 | -0.231 | 0.013 | -0.006 | -0.026 | -0.046 | -0.071 | -0.093 | -0.109 | -0.012 | 0.039 | -0.011 |
|  | (0.012) | (0.001) | (0.018) | (0.001) | (0.003) | (0.003) | (0.003) | (0.003) | (0.003) | (0.003) | (0.002) | (0.002) | (0.003) |
| Massachusetts | 0.554 | 0.021 | -0.207 | 0.011 | -0.014 | -0.033 | -0.059 | -0.087 | -0.113 | -0.121 | -0.015 | 0.096 | 0.008 |
|  | (0.014) | (0.001) | (0.022) | (0.001) | (0.003) | (0.003) | (0.003) | (0.003) | (0.003) | (0.003) | (0.002) | (0.003) | (0.003) |
| Michigan | 0.641 | 0.016 | -0.309 | 0.017 | -0.018 | -0.036 | -0.061 | -0.084 | -0.105 | -0.109 | -0.010 | 0.091 | -0.019 |
|  | (0.015) | (0.001) | (0.024) | (0.001) | (0.004) | (0.004) | (0.004) | (0.004) | (0.004) | (0.004) | (0.002) | (0.003) | (0.003) |
| Minnesota | 0.713 | 0.012 | -0.211 | 0.011 | -0.009 | -0.020 | -0.038 | -0.054 | -0.072 | -0.084 | -0.002 | 0.077 | -0.002 |
|  | (0.013) | (0.001) | (0.018) | (0.001) | (0.003) | (0.003) | (0.003) | (0.003) | (0.003) | (0.003) | (0.002) | (0.003) | (0.002) |
| Mississippi | 0.448 | 0.030 | -0.137 | 0.006 | -0.031 | -0.064 | -0.105 | -0.150 | -0.190 | -0.214 | -0.025 | 0.078 | 0.002 |
|  | (0.018) | (0.001) | (0.028) | (0.002) | (0.005) | (0.005) | (0.005) | (0.005) | (0.005) | (0.005) | (0.003) | (0.003) | (0.005) |
| Missouri | 0.568 | 0.022 | -0.274 | 0.013 | -0.020 | -0.043 | -0.077 | -0.109 | -0.131 | -0.142 | -0.015 | 0.078 | 0.004 |
|  | (0.017) | (0.001) | (0.027) | (0.002) | (0.005) | (0.005) | (0.005) | (0.005) | (0.005) | (0.005) | (0.003) | (0.004) | (0.004) |
| Montana | 0.615 | 0.017 | -0.181 | 0.009 | -0.022 | -0.042 | -0.066 | -0.093 | -0.117 | -0.125 | -0.009 | 0.116 | 0.001 |
|  | (0.019) | (0.001) | (0.029) | (0.002) | (0.004) | (0.004) | (0.005) | (0.004) | (0.005) | (0.005) | (0.003) | (0.005) | (0.004) |
| Nebraska | 0.634 | 0.016 | -0.144 | 0.008 | -0.013 | -0.033 | -0.051 | -0.067 | -0.093 | -0.106 | -0.008 | 0.105 | -0.009 |
|  | (0.015) | (0.001) | (0.021) | (0.001) | (0.003) | (0.003) | (0.003) | (0.003) | (0.003) | (0.003) | (0.002) | (0.004) | (0.002) |
| Nevada | 0.654 | 0.017 | -0.220 | 0.011 | -0.019 | -0.031 | -0.070 | -0.089 | -0.113 | -0.126 | -0.018 | 0.070 | 0.003 |
|  | (0.019) | (0.001) | (0.031) | (0.002) | (0.005) | (0.005) | (0.006) | (0.006) | (0.006) | (0.006) | (0.003) | (0.004) | (0.005) |
| New Hampshire | 0.725 | 0.013 | -0.352 | 0.020 | -0.019 | -0.026 | -0.046 | -0.064 | -0.090 | -0.089 | -0.011 | 0.056 | -0.020 |
|  | (0.018) | (0.001) | (0.027) | (0.002) | (0.004) | (0.004) | (0.004) | (0.004) | (0.004) | (0.005) | (0.002) | (0.006) | (0.004) |
| New Jersey | 0.619 | 0.018 | -0.200 | 0.011 | -0.022 | -0.035 | -0.065 | -0.088 | -0.110 | -0.134 | -0.015 | 0.096 | -0.016 |
|  | (0.015) | (0.001) | (0.024) | (0.001) | (0.003) | (0.003) | (0.003) | (0.003) | (0.004) | (0.004) | (0.002) | (0.002) | (0.004) |
| New Mexico | 0.553 | 0.025 | -0.118 | 0.005 | -0.026 | -0.056 | -0.089 | -0.114 | -0.146 | -0.161 | -0.016 | 0.069 | -0.009 |
|  | (0.016) | (0.001) | (0.025) | (0.002) | (0.005) | (0.005) | (0.005) | (0.005) | (0.005) | (0.005) | (0.003) | (0.003) | (0.004) |
| New York | 0.688 | 0.015 | -0.234 | 0.012 | -0.020 | -0.040 | -0.063 | -0.090 | -0.117 | -0.124 | -0.014 | 0.078 | -0.006 |
|  | (0.012) | (0.001) | (0.017) | (0.001) | (0.003) | (0.003) | (0.003) | (0.004) | (0.004) | (0.004) | (0.002) | (0.002) | (0.003) |
| North Carolina | 0.523 | 0.026 | -0.193 | 0.009 | -0.021 | -0.048 | -0.085 | -0.117 | -0.149 | -0.155 | -0.013 | 0.059 | 0.002 |
|  | (0.014) | (0.001) | (0.023) | (0.001) | (0.003) | (0.003) | (0.004) | (0.004) | (0.004) | (0.004) | (0.002) | (0.003) | (0.004) |
| North Dakota | 0.623 | 0.014 | -0.087 | 0.005 | -0.012 | -0.030 | -0.049 | -0.070 | -0.091 | -0.115 | -0.000 | 0.122 | -0.023 |
|  | (0.020) | (0.001) | (0.029) | (0.002) | (0.004) | (0.004) | (0.004) | (0.004) | (0.005) | (0.005) | (0.003) | (0.007) | (0.004) |
| Ohio | 0.616 | 0.018 | -0.325 | 0.017 | -0.005 | -0.030 | -0.055 | -0.084 | -0.107 | -0.124 | -0.006 | 0.089 | -0.003 |
|  | (0.016) | (0.001) | (0.023) | (0.001) | (0.004) | (0.004) | (0.004) | (0.004) | (0.004) | (0.004) | (0.002) | (0.003) | (0.003) |
| Oklahoma | 0.600 | 0.021 | -0.285 | 0.015 | -0.014 | -0.047 | -0.082 | -0.119 | -0.148 | -0.165 | -0.010 | 0.063 | 0.010 |
|  | (0.016) | (0.001) | (0.027) | (0.002) | (0.004) | (0.004) | (0.005) | (0.005) | (0.005) | (0.005) | (0.003) | (0.003) | (0.004) |
| Oregon | 0.594 | 0.020 | -0.193 | 0.009 | -0.012 | -0.033 | -0.067 | -0.080 | -0.107 | -0.106 | -0.008 | 0.065 | -0.012 |
|  | (0.016) | (0.001) | (0.026) | (0.002) | (0.004) | (0.005) | (0.005) | (0.005) | (0.005) | (0.005) | (0.003) | (0.004) | (0.004) |
| Pennsylvania | 0.604 | 0.018 | -0.241 | 0.012 | -0.010 | -0.031 | -0.055 | -0.084 | -0.113 | -0.115 | -0.009 | 0.092 | -0.004 |
|  | (0.013) | (0.001) | (0.022) | (0.001) | (0.003) | (0.003) | (0.004) | (0.004) | (0.004) | (0.004) | (0.002) | (0.003) | (0.003) |
| Rhode Island | 0.549 | 0.021 | -0.171 | 0.009 | -0.009 | -0.039 | -0.057 | -0.090 | -0.110 | -0.116 | -0.015 | 0.103 | -0.019 |
|  | (0.017) | (0.001) | (0.027) | (0.002) | (0.004) | (0.005) | (0.005) | (0.005) | (0.005) | (0.005) | (0.003) | (0.004) | (0.004) |
| South Carolina | 0.613 | 0.021 | -0.294 | 0.016 | -0.024 | -0.050 | -0.083 | -0.118 | -0.156 | -0.158 | -0.018 | 0.062 | -0.004 |
|  | (0.014) | (0.001) | (0.022) | (0.001) | (0.004) | (0.004) | (0.004) | (0.004) | (0.004) | (0.004) | (0.002) | (0.003) | (0.003) |
| South Dakota | 0.669 | 0.011 | -0.167 | 0.010 | -0.010 | -0.028 | -0.051 | -0.070 | -0.102 | -0.111 | 0.000 | 0.134 | -0.017 |
|  | (0.015) | (0.001) | (0.023) | (0.002) | (0.004) | (0.004) | (0.004) | (0.004) | (0.004) | (0.004) | (0.002) | (0.004) | (0.004) |
| Tennessee | 0.487 | 0.031 | -0.234 | 0.010 | -0.027 | -0.059 | -0.103 | -0.138 | -0.164 | -0.176 | -0.015 | 0.034 | 0.004 |
|  | (0.016) | (0.001) | (0.025) | (0.002) | (0.005) | (0.005) | (0.005) | (0.005) | (0.005) | (0.006) | (0.003) | (0.004) | (0.005) |
| Texas | 0.469 | 0.028 | 0.001 | -0.002 | -0.019 | -0.044 | -0.076 | -0.116 | -0.150 | -0.171 | -0.017 | 0.076 | -0.008 |
|  | (0.013) | (0.001) | (0.019) | (0.001) | (0.004) | (0.004) | (0.004) | (0.004) | (0.004) | (0.004) | (0.002) | (0.002) | (0.003) |
| Utah | 0.664 | 0.014 | -0.148 | 0.009 | -0.015 | -0.027 | -0.051 | -0.073 | -0.095 | -0.106 | -0.008 | 0.084 | -0.009 |
|  | (0.016) | (0.001) | (0.022) | (0.001) | (0.003) | (0.003) | (0.003) | (0.003) | (0.004) | (0.004) | (0.002) | (0.004) | (0.003) |
| Vermont | 0.609 | 0.018 | -0.187 | 0.010 | -0.013 | -0.025 | -0.043 | -0.061 | -0.078 | -0.090 | -0.003 | 0.085 | -0.012 |
|  | (0.015) | (0.001) | (0.024) | (0.001) | (0.004) | (0.004) | (0.004) | (0.004) | (0.004) | (0.004) | (0.002) | (0.006) | (0.004) |
| Virginia | 0.590 | 0.023 | -0.209 | 0.010 | -0.017 | -0.034 | -0.057 | -0.082 | -0.114 | -0.125 | -0.016 | 0.041 | -0.013 |
|  | (0.015) | (0.001) | (0.023) | (0.001) | (0.004) | (0.004) | (0.004) | (0.004) | (0.004) | (0.004) | (0.002) | (0.003) | (0.003) |
| Washington | 0.636 | 0.018 | -0.210 | 0.011 | -0.012 | -0.034 | -0.057 | -0.081 | -0.105 | -0.106 | -0.008 | 0.065 | 0.000 |
|  | (0.012) | (0.001) | (0.019) | (0.001) | (0.003) | (0.003) | (0.003) | (0.003) | (0.003) | (0.003) | (0.002) | (0.003) | (0.003) |
| West Virginia | 0.389 | 0.035 | -0.183 | 0.008 | -0.034 | -0.064 | -0.110 | -0.136 | -0.179 | -0.180 | 0.001 | 0.051 | -0.007 |
|  | (0.018) | (0.001) | (0.026) | (0.002) | (0.006) | (0.006) | (0.006) | (0.006) | (0.006) | (0.006) | (0.003) | (0.007) | (0.005) |
| Wisconsin | 0.672 | 0.013 | -0.228 | 0.012 | -0.019 | -0.036 | -0.051 | -0.071 | -0.096 | -0.107 | -0.002 | 0.119 | -0.005 |
|  | (0.017) | (0.001) | (0.027) | (0.002) | (0.004) | (0.004) | (0.004) | (0.004) | (0.005) | (0.005) | (0.003) | (0.004) | (0.004) |
| Wyoming | 0.695 | 0.013 | -0.227 | 0.012 | -0.021 | -0.023 | -0.041 | -0.064 | -0.091 | -0.102 | -0.008 | 0.080 | -0.006 |
|  | (0.018) | (0.001) | (0.029) | (0.002) | (0.005) | (0.004) | (0.005) | (0.005) | (0.005) | (0.005) | (0.003) | (0.006) | (0.005) |

*Note:* Sample size = 4,904,293. The state-stratified regression models for this table are the same as those for Table 2 in the manuscript but the latter reported only the coefficients for the intercept, education, year, and the education-by-year interaction.

**Figure S1**

*Estimated Probability of Favorable Health for Adults Ages 30–64 with a High School Credential versus a Bachelor’s or Higher, by State, 1993–2019*


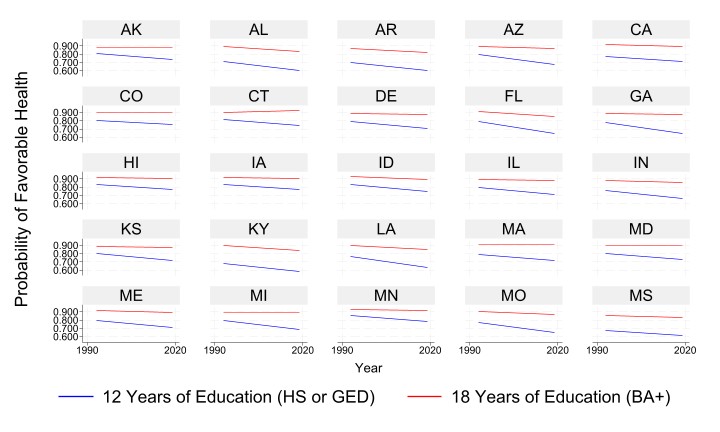


**High School Credential**

**Bachelor’s Degree or Higher**


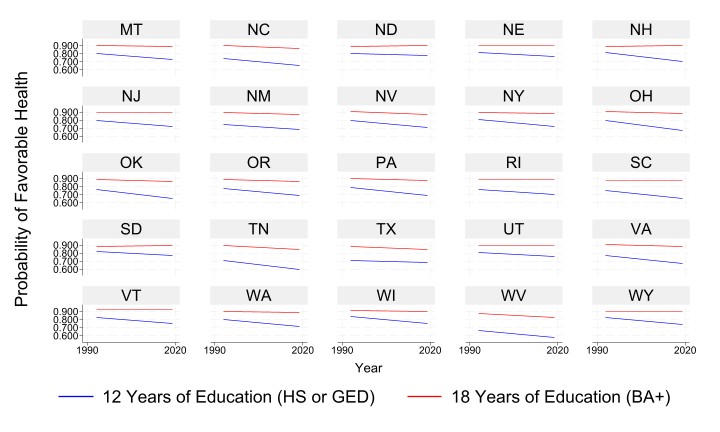


**High School Credential**

**Bachelor’s Degree or Higher**

*Note:* Probabilities are estimated from state-stratified models including education, year, education-by-year interaction, age, sex, race and sampling frame. The probabilities are for women ages 54–59 who are non-Hispanic White.

| **Table S3**  *Summary of Variables Across the 50 States (N=50) Among Adults Who Were Able to Work* | | | | | | | |
| --- | --- | --- | --- | --- | --- | --- | --- |
|  | 1994 | | |  | 2019 | | |
| Variable | Average | Min | Max |  | Average | Min | Max |
|  |  |  |  |  |  |  |  |
| Favorable self-rated health | .908 | .840 | .941 |  | .871 | .824 | .922 |
| Educational attainment (years) | 13.932 | 12.212 | 14.914 |  | 15.032 | 14.345 | 15.802 |
|  |  |  |  |  |  |  |  |
| Individual-level factors |  |  |  |  |  |  |  |
| Household income (1000$) | 44.678 | 21.936 | 62.368 |  | 72.051 | 62.260 | 80.777 |
| Currently employed | .804 | .704 | .873 |  | .800 | .739 | .863 |
| Has healthcare coverage | .876 | .803 | .937 |  | .890 | .779 | .949 |
| Currently married | .655 | .580 | .724 |  | .627 | .563 | .772 |
| Current smoker | .260 | .173 | .328 |  | .169 | .081 | .242 |
| Obese | .164 | .106 | .211 |  | .359 | .267 | .476 |
|  |  |  |  |  |  |  |  |
| State-level contextual factors |  |  |  |  |  |  |  |
| Bachelor’s or higher | .284 | .161 | .413 |  | .311 | .202 | .431 |
| Immigrants | .057 | .010 | .235 |  | .092 | .016 | .267 |
| Policy context score (index) | -0.012 | -2.104 | 1.848 |  | 0.025 | -3.479 | 2.912 |
|  |  |  |  |  |  |  |  |
| Demographics |  |  |  |  |  |  |  |
| Age |  |  |  |  |  |  |  |
| 30-34 years | .197 | .158 | .240 |  | .113 | .078 | .152 |
| 35-39 years | .208 | .154 | .246 |  | .124 | .086 | .164 |
| 40-44 years | .178 | .139 | .211 |  | .120 | .092 | .162 |
| 45-49 years | .143 | .115 | .177 |  | .127 | .102 | .146 |
| 50-54 years | .108 | .081 | .145 |  | .146 | .125 | .173 |
| 55-59 years | .086 | .070 | .112 |  | .173 | .128 | .212 |
| 60-64 years | .080 | .046 | .114 |  | .198 | .136 | .259 |
| Sex |  |  |  |  |  |  |  |
| Female | .560 | .501 | .635 |  | .515 | .477 | .569 |
| Male | .440 | .365 | .499 |  | .485 | .431 | .523 |
| Race |  |  |  |  |  |  |  |
| Non-Hispanic White | .840 | .397 | .976 |  | .768 | .317 | .954 |
| Other | .160 | .024 | .603 |  | .230 | .046 | .684 |

*Note.* All numbers are proportions unless noted otherwise. The values in the cells represent the average, minimum and maximum value for each variable among the 50 values for each state in that year.

| **Table S4**  *Regression Coefficients (and Standard Errors) Predicting the Probability of Reporting Favorable Health among Adults Ages 30–64 Years, by U.S. State, 1994–2019, Among Adults Who Were Able to Work* | | | | | | | | | | | | | | |
| --- | --- | --- | --- | --- | --- | --- | --- | --- | --- | --- | --- | --- | --- | --- |
| State | Intercept | Education | Year | Education-by-year interaction | Age  35-39 | Age  40-44 | Age  45-49 | Age  50-54 | Age  55-59 | Age  60-64 | Female | Non-Hispanic white | Cellphone survey mode | p-value for interaction |
| Alabama | 0.587 | 0.021 | -0.043 | 0.002 | -0.013 | -0.035 | -0.051 | -0.074 | -0.103 | -0.130 | -0.007 | 0.061 | -0.006 | .364 |
|  | (0.021) | (0.001) | (0.032) | (0.002) | (0.005) | (0.005) | (0.005) | (0.005) | (0.006) | (0.006) | (0.003) | (0.004) | (0.005) |  |
| Alaska | 0.743 | 0.011 | -0.083 | 0.004 | -0.015 | -0.022 | -0.044 | -0.051 | -0.070 | -0.087 | -0.008 | 0.057 | -0.005 | .049 |
|  | (0.020) | (0.001) | (0.033) | (0.002) | (0.005) | (0.005) | (0.005) | (0.005) | (0.006) | (0.006) | (0.003) | (0.004) | (0.005) |  |
| Arizona | 0.698 | 0.014 | -0.197 | 0.011 | -0.009 | -0.037 | -0.048 | -0.062 | -0.076 | -0.095 | -0.005 | 0.066 | -0.003 | <.001 |
|  | (0.020) | (0.001) | (0.030) | (0.002) | (0.005) | (0.005) | (0.005) | (0.005) | (0.005) | (0.005) | (0.003) | (0.003) | (0.004) |  |
| Arkansas | 0.579 | 0.021 | -0.051 | 0.002 | -0.011 | -0.032 | -0.054 | -0.076 | -0.099 | -0.126 | -0.002 | 0.069 | -0.008 | .394 |
|  | (0.022) | (0.001) | (0.036) | (0.002) | (0.005) | (0.006) | (0.006) | (0.006) | (0.006) | (0.006) | (0.003) | (0.005) | (0.006) |  |
| California | 0.638 | 0.018 | -0.188 | 0.010 | -0.009 | -0.028 | -0.049 | -0.066 | -0.079 | -0.092 | -0.009 | 0.078 | -0.005 | <.001 |
|  | (0.013) | (0.001) | (0.020) | (0.001) | (0.003) | (0.003) | (0.004) | (0.004) | (0.004) | (0.004) | (0.002) | (0.002) | (0.003) |  |
| Colorado | 0.677 | 0.014 | -0.064 | 0.004 | -0.012 | -0.019 | -0.032 | -0.038 | -0.051 | -0.063 | -0.003 | 0.075 | -0.017 | .009 |
|  | (0.017) | (0.001) | (0.025) | (0.002) | (0.003) | (0.003) | (0.003) | (0.003) | (0.003) | (0.004) | (0.002) | (0.003) | (0.003) |  |
| Connecticut | 0.752 | 0.010 | -0.168 | 0.011 | -0.007 | -0.021 | -0.029 | -0.041 | -0.048 | -0.069 | -0.004 | 0.068 | -0.018 | <.001 |
|  | (0.016) | (0.001) | (0.025) | (0.002) | (0.004) | (0.004) | (0.004) | (0.004) | (0.004) | (0.004) | (0.002) | (0.003) | (0.003) |  |
| Delaware | 0.739 | 0.012 | -0.186 | 0.011 | -0.002 | -0.014 | -0.040 | -0.048 | -0.068 | -0.085 | -0.005 | 0.051 | -0.015 | <.001 |
|  | (0.019) | (0.001) | (0.033) | (0.002) | (0.005) | (0.005) | (0.005) | (0.005) | (0.006) | (0.006) | (0.003) | (0.004) | (0.005) |  |
| Florida | 0.684 | 0.015 | -0.197 | 0.010 | -0.012 | -0.025 | -0.042 | -0.061 | -0.078 | -0.096 | -0.001 | 0.054 | -0.001 | <.001 |
|  | (0.014) | (0.001) | (0.021) | (0.001) | (0.003) | (0.003) | (0.003) | (0.003) | (0.004) | (0.004) | (0.002) | (0.002) | (0.003) |  |
| Georgia | 0.783 | 0.011 | -0.316 | 0.018 | -0.011 | -0.028 | -0.045 | -0.064 | -0.089 | -0.110 | -0.006 | 0.038 | -0.009 | <.001 |
|  | (0.014) | (0.001) | (0.025) | (0.002) | (0.004) | (0.004) | (0.004) | (0.004) | (0.005) | (0.005) | (0.002) | (0.003) | (0.004) |  |
| Hawaii | 0.768 | 0.010 | -0.124 | 0.006 | -0.013 | -0.024 | -0.036 | -0.058 | -0.066 | -0.078 | 0.001 | 0.065 | -0.008 | <.001 |
|  | (0.018) | (0.001) | (0.028) | (0.002) | (0.004) | (0.004) | (0.004) | (0.004) | (0.004) | (0.005) | (0.002) | (0.002) | (0.004) |  |
| Idaho | 0.710 | 0.012 | -0.123 | 0.006 | -0.010 | -0.016 | -0.038 | -0.038 | -0.054 | -0.077 | 0.002 | 0.067 | 0.006 | <.001 |
|  | (0.016) | (0.001) | (0.027) | (0.002) | (0.004) | (0.004) | (0.004) | (0.004) | (0.004) | (0.005) | (0.002) | (0.005) | (0.004) |  |
| Illinois | 0.706 | 0.012 | -0.180 | 0.010 | -0.007 | -0.013 | -0.030 | -0.045 | -0.070 | -0.077 | -0.005 | 0.078 | -0.016 | <.001 |
|  | (0.017) | (0.001) | (0.027) | (0.002) | (0.004) | (0.004) | (0.004) | (0.004) | (0.005) | (0.005) | (0.002) | (0.003) | (0.004) |  |
| Indiana | 0.652 | 0.015 | -0.114 | 0.006 | -0.004 | -0.018 | -0.031 | -0.043 | -0.063 | -0.080 | -0.006 | 0.072 | -0.018 | <.001 |
|  | (0.017) | (0.001) | (0.026) | (0.002) | (0.004) | (0.004) | (0.004) | (0.004) | (0.004) | (0.005) | (0.002) | (0.004) | (0.004) |  |
| Iowa | 0.722 | 0.010 | -0.113 | 0.006 | 0.002 | -0.015 | -0.025 | -0.033 | -0.044 | -0.067 | 0.001 | 0.087 | -0.010 | <.001 |
|  | (0.015) | (0.001) | (0.023) | (0.001) | (0.003) | (0.004) | (0.004) | (0.004) | (0.004) | (0.004) | (0.002) | (0.006) | (0.003) |  |
| Kansas | 0.710 | 0.012 | -0.088 | 0.005 | -0.009 | -0.019 | -0.031 | -0.042 | -0.052 | -0.076 | -0.001 | 0.064 | -0.023 | .001 |
|  | (0.016) | (0.001) | (0.024) | (0.001) | (0.003) | (0.003) | (0.003) | (0.003) | (0.003) | (0.003) | (0.002) | (0.003) | (0.003) |  |
| Kentucky | 0.498 | 0.027 | 0.007 | -0.002 | -0.006 | -0.024 | -0.052 | -0.070 | -0.104 | -0.132 | -0.013 | 0.044 | 0.006 | .174 |
|  | (0.017) | (0.001) | (0.026) | (0.002) | (0.004) | (0.004) | (0.005) | (0.005) | (0.005) | (0.005) | (0.003) | (0.005) | (0.004) |  |
| Louisiana | 0.698 | 0.016 | -0.192 | 0.009 | -0.017 | -0.038 | -0.055 | -0.085 | -0.111 | -0.142 | -0.013 | 0.059 | 0.014 | <.001 |
|  | (0.019) | (0.001) | (0.032) | (0.002) | (0.004) | (0.005) | (0.005) | (0.005) | (0.005) | (0.005) | (0.003) | (0.003) | (0.005) |  |
| Maine | 0.697 | 0.013 | -0.077 | 0.004 | -0.003 | -0.007 | -0.020 | -0.033 | -0.037 | -0.054 | 0.006 | 0.056 | -0.018 | .010 |
|  | (0.019) | (0.001) | (0.026) | (0.002) | (0.004) | (0.004) | (0.004) | (0.004) | (0.004) | (0.004) | (0.002) | (0.007) | (0.003) |  |
| Maryland | 0.717 | 0.013 | -0.083 | 0.005 | -0.003 | -0.014 | -0.027 | -0.041 | -0.056 | -0.077 | -0.002 | 0.038 | -0.012 | <.001 |
|  | (0.013) | (0.001) | (0.021) | (0.001) | (0.003) | (0.003) | (0.003) | (0.003) | (0.003) | (0.004) | (0.002) | (0.002) | (0.003) |  |
| Massachusetts | 0.673 | 0.014 | -0.119 | 0.007 | -0.006 | -0.015 | -0.032 | -0.046 | -0.060 | -0.077 | -0.005 | 0.080 | -0.003 | <.001 |
|  | (0.016) | (0.001) | (0.026) | (0.002) | (0.003) | (0.003) | (0.003) | (0.003) | (0.003) | (0.003) | (0.002) | (0.003) | (0.003) |  |
| Michigan | 0.687 | 0.014 | -0.115 | 0.006 | -0.009 | -0.019 | -0.034 | -0.044 | -0.062 | -0.077 | 0.001 | 0.070 | -0.016 | <.001 |
|  | (0.016) | (0.001) | (0.025) | (0.002) | (0.004) | (0.004) | (0.004) | (0.004) | (0.004) | (0.004) | (0.002) | (0.003) | (0.003) |  |
| Minnesota | 0.756 | 0.010 | -0.125 | 0.006 | -0.006 | -0.011 | -0.021 | -0.031 | -0.043 | -0.062 | 0.003 | 0.064 | -0.005 | <.001 |
|  | (0.013) | (0.001) | (0.019) | (0.001) | (0.003) | (0.003) | (0.003) | (0.003) | (0.003) | (0.003) | (0.002) | (0.004) | (0.002) |  |
| Mississippi | 0.555 | 0.022 | 0.004 | -0.000 | -0.017 | -0.035 | -0.060 | -0.083 | -0.105 | -0.142 | -0.014 | 0.071 | -0.007 | .839 |
|  | (0.021) | (0.001) | (0.034) | (0.002) | (0.005) | (0.005) | (0.005) | (0.005) | (0.006) | (0.006) | (0.003) | (0.003) | (0.005) |  |
| Missouri | 0.645 | 0.017 | -0.075 | 0.004 | -0.011 | -0.023 | -0.036 | -0.057 | -0.074 | -0.094 | -0.005 | 0.060 | -0.005 | .058 |
|  | (0.019) | (0.001) | (0.030) | (0.002) | (0.004) | (0.005) | (0.005) | (0.005) | (0.005) | (0.005) | (0.003) | (0.005) | (0.004) |  |
| Montana | 0.712 | 0.011 | -0.129 | 0.007 | -0.014 | -0.028 | -0.040 | -0.054 | -0.068 | -0.083 | -0.002 | 0.096 | -0.002 | .001 |
|  | (0.020) | (0.001) | (0.030) | (0.002) | (0.004) | (0.004) | (0.004) | (0.004) | (0.004) | (0.005) | (0.002) | (0.005) | (0.004) |  |
| Nebraska | 0.706 | 0.011 | -0.111 | 0.007 | -0.008 | -0.017 | -0.026 | -0.034 | -0.050 | -0.068 | 0.000 | 0.094 | -0.012 | <.001 |
|  | (0.016) | (0.001) | (0.022) | (0.001) | (0.003) | (0.003) | (0.003) | (0.003) | (0.003) | (0.003) | (0.002) | (0.004) | (0.002) |  |
| Nevada | 0.670 | 0.015 | -0.118 | 0.006 | -0.013 | -0.016 | -0.045 | -0.056 | -0.069 | -0.087 | -0.005 | 0.067 | -0.004 | .009 |
|  | (0.021) | (0.001) | (0.035) | (0.002) | (0.005) | (0.005) | (0.006) | (0.006) | (0.006) | (0.006) | (0.003) | (0.004) | (0.005) |  |
| New Hampshire | 0.772 | 0.010 | -0.127 | 0.007 | -0.007 | -0.007 | -0.019 | -0.030 | -0.045 | -0.055 | 0.001 | 0.036 | -0.015 | <.001 |
|  | (0.018) | (0.001) | (0.028) | (0.002) | (0.004) | (0.004) | (0.004) | (0.004) | (0.004) | (0.004) | (0.002) | (0.006) | (0.004) |  |
| New Jersey | 0.683 | 0.014 | -0.128 | 0.007 | -0.014 | -0.021 | -0.044 | -0.056 | -0.070 | -0.096 | -0.006 | 0.087 | -0.016 | <.001 |
|  | (0.017) | (0.001) | (0.028) | (0.002) | (0.003) | (0.003) | (0.003) | (0.003) | (0.004) | (0.004) | (0.002) | (0.002) | (0.004) |  |
| New Mexico | 0.629 | 0.020 | -0.100 | 0.005 | -0.018 | -0.034 | -0.058 | -0.071 | -0.091 | -0.111 | -0.013 | 0.066 | -0.009 | .011 |
|  | (0.018) | (0.001) | (0.029) | (0.002) | (0.005) | (0.005) | (0.005) | (0.005) | (0.005) | (0.005) | (0.003) | (0.003) | (0.004) |  |
| New York | 0.738 | 0.010 | -0.147 | 0.009 | -0.014 | -0.024 | -0.037 | -0.052 | -0.067 | -0.086 | -0.004 | 0.076 | -0.011 | <.001 |
|  | (0.014) | (0.001) | (0.019) | (0.001) | (0.003) | (0.003) | (0.004) | (0.004) | (0.004) | (0.004) | (0.002) | (0.003) | (0.003) |  |
| North Carolina | 0.626 | 0.018 | -0.124 | 0.007 | -0.011 | -0.024 | -0.046 | -0.059 | -0.078 | -0.093 | -0.004 | 0.058 | -0.011 | <.001 |
|  | (0.016) | (0.001) | (0.028) | (0.002) | (0.003) | (0.003) | (0.004) | (0.004) | (0.004) | (0.004) | (0.002) | (0.003) | (0.004) |  |
| North Dakota | 0.713 | 0.010 | -0.062 | 0.004 | -0.010 | -0.022 | -0.037 | -0.045 | -0.064 | -0.088 | 0.004 | 0.096 | -0.022 | .029 |
|  | (0.020) | (0.001) | (0.030) | (0.002) | (0.004) | (0.004) | (0.004) | (0.004) | (0.005) | (0.005) | (0.003) | (0.007) | (0.004) |  |
| Ohio | 0.676 | 0.014 | -0.130 | 0.007 | 0.002 | -0.013 | -0.024 | -0.040 | -0.061 | -0.086 | 0.002 | 0.070 | -0.009 | <.001 |
|  | (0.017) | (0.001) | (0.024) | (0.002) | (0.004) | (0.004) | (0.004) | (0.004) | (0.004) | (0.004) | (0.002) | (0.004) | (0.003) |  |
| Oklahoma | 0.670 | 0.015 | -0.144 | 0.009 | -0.007 | -0.027 | -0.043 | -0.059 | -0.079 | -0.108 | -0.002 | 0.055 | -0.011 | <.001 |
|  | (0.018) | (0.001) | (0.030) | (0.002) | (0.004) | (0.004) | (0.005) | (0.005) | (0.005) | (0.005) | (0.003) | (0.003) | (0.004) |  |
| Oregon | 0.663 | 0.015 | -0.121 | 0.006 | -0.003 | -0.015 | -0.038 | -0.041 | -0.057 | -0.069 | 0.000 | 0.058 | -0.013 | .001 |
|  | (0.018) | (0.001) | (0.030) | (0.002) | (0.004) | (0.005) | (0.005) | (0.005) | (0.005) | (0.005) | (0.003) | (0.005) | (0.004) |  |
| Pennsylvania | 0.696 | 0.013 | -0.119 | 0.006 | -0.003 | -0.017 | -0.030 | -0.044 | -0.070 | -0.083 | -0.003 | 0.073 | -0.009 | <.001 |
|  | (0.014) | (0.001) | (0.023) | (0.001) | (0.003) | (0.003) | (0.003) | (0.004) | (0.004) | (0.004) | (0.002) | (0.003) | (0.003) |  |
| Rhode Island | 0.636 | 0.015 | -0.074 | 0.005 | -0.002 | -0.018 | -0.030 | -0.041 | -0.059 | -0.068 | -0.006 | 0.103 | -0.020 | .012 |
|  | (0.020) | (0.001) | (0.031) | (0.002) | (0.004) | (0.004) | (0.005) | (0.005) | (0.005) | (0.005) | (0.002) | (0.005) | (0.004) |  |
| South Carolina | 0.681 | 0.015 | -0.118 | 0.007 | -0.014 | -0.027 | -0.044 | -0.063 | -0.087 | -0.105 | -0.006 | 0.053 | -0.007 | <.001 |
|  | (0.017) | (0.001) | (0.026) | (0.002) | (0.004) | (0.004) | (0.004) | (0.004) | (0.004) | (0.004) | (0.002) | (0.003) | (0.003) |  |
| South Dakota | 0.720 | 0.009 | -0.090 | 0.006 | -0.008 | -0.019 | -0.033 | -0.047 | -0.070 | -0.081 | 0.004 | 0.114 | -0.015 | <.001 |
|  | (0.015) | (0.001) | (0.025) | (0.002) | (0.004) | (0.004) | (0.004) | (0.004) | (0.004) | (0.004) | (0.002) | (0.005) | (0.004) |  |
| Tennessee | 0.585 | 0.022 | -0.063 | 0.003 | -0.014 | -0.037 | -0.059 | -0.080 | -0.094 | -0.121 | -0.011 | 0.041 | -0.012 | .118 |
|  | (0.021) | (0.001) | (0.033) | (0.002) | (0.005) | (0.005) | (0.006) | (0.006) | (0.006) | (0.006) | (0.003) | (0.005) | (0.005) |  |
| Texas | 0.530 | 0.023 | 0.033 | -0.002 | -0.013 | -0.030 | -0.049 | -0.071 | -0.095 | -0.121 | -0.011 | 0.077 | -0.013 | .138 |
|  | (0.015) | (0.001) | (0.023) | (0.001) | (0.004) | (0.004) | (0.004) | (0.004) | (0.004) | (0.004) | (0.002) | (0.002) | (0.003) |  |
| Utah | 0.743 | 0.009 | -0.130 | 0.008 | -0.011 | -0.021 | -0.032 | -0.041 | -0.057 | -0.072 | 0.000 | 0.077 | -0.011 | <.001 |
|  | (0.016) | (0.001) | (0.023) | (0.001) | (0.003) | (0.003) | (0.003) | (0.003) | (0.004) | (0.004) | (0.002) | (0.004) | (0.003) |  |
| Vermont | 0.699 | 0.014 | -0.024 | 0.001 | -0.006 | -0.012 | -0.023 | -0.034 | -0.044 | -0.059 | 0.005 | 0.048 | -0.010 | .402 |
|  | (0.016) | (0.001) | (0.025) | (0.002) | (0.003) | (0.003) | (0.003) | (0.004) | (0.004) | (0.004) | (0.002) | (0.006) | (0.003) |  |
| Virginia | 0.700 | 0.015 | -0.112 | 0.006 | -0.013 | -0.021 | -0.035 | -0.050 | -0.069 | -0.091 | -0.006 | 0.043 | -0.016 | <.001 |
|  | (0.017) | (0.001) | (0.026) | (0.002) | (0.004) | (0.004) | (0.004) | (0.004) | (0.004) | (0.004) | (0.002) | (0.003) | (0.003) |  |
| Washington | 0.717 | 0.012 | -0.146 | 0.008 | -0.008 | -0.021 | -0.030 | -0.041 | -0.058 | -0.074 | 0.001 | 0.060 | -0.008 | <.001 |
|  | (0.013) | (0.001) | (0.022) | (0.001) | (0.003) | (0.003) | (0.003) | (0.003) | (0.003) | (0.003) | (0.002) | (0.003) | (0.003) |  |
| West Virginia | 0.531 | 0.025 | 0.008 | -0.000 | -0.021 | -0.036 | -0.058 | -0.071 | -0.107 | -0.125 | -0.005 | 0.039 | -0.023 | .815 |
|  | (0.021) | (0.001) | (0.032) | (0.002) | (0.005) | (0.006) | (0.006) | (0.006) | (0.006) | (0.006) | (0.003) | (0.007) | (0.005) |  |
| Wisconsin | 0.712 | 0.011 | -0.123 | 0.006 | -0.016 | -0.025 | -0.034 | -0.049 | -0.062 | -0.079 | 0.002 | 0.094 | 0.001 | .001 |
|  | (0.018) | (0.001) | (0.028) | (0.002) | (0.004) | (0.004) | (0.004) | (0.004) | (0.005) | (0.005) | (0.002) | (0.005) | (0.004) |  |
| Wyoming | 0.766 | 0.009 | -0.156 | 0.009 | -0.015 | -0.011 | -0.024 | -0.033 | -0.054 | -0.067 | -0.002 | 0.059 | -0.005 | <.001 |
|  | (0.018) | (0.001) | (0.029) | (0.002) | (0.004) | (0.004) | (0.004) | (0.004) | (0.005) | (0.005) | (0.002) | (0.006) | (0.005) |  |

| **Table S5.**  *Values Shown on the Box Plots in Figure 3 in the Main Manuscript for Each of the Nine Factors* | | | | | | | | | |
| --- | --- | --- | --- | --- | --- | --- | --- | --- | --- |
|  | Income | Employment | Health care | Marriage | Obesity | Smoking | College Graduates | Immigrants | Policy Contexts |
| Lower whisker | 0.6 | -3.8 | -4.0 | -1.8 | -3.0 | -9.0 | -1.5 | -4.5 | -3.3 |
| First quartile | 10.2 | 3.4 | 0.1 | 0.7 | 3.5 | -3.5 | 1.2 | -0.4 | -0.5 |
| Median | 14.5 | 6.5 | 0.9 | 2.2 | 5.9 | -1.1 | 2.5 | 1.6 | 0.2 |
| Third quartile | 22.1 | 9.4 | 3.0 | 3.1 | 8.9 | 2.8 | 3.4 | 3.6 | 1.4 |
| Upper whisker | 37.6 | 15.2 | 6.4 | 5.6 | 16.2 | 9.9 | 5.7 | 6.1 | 4.2 |

**Figure S2**

*Contribution of Each Factor to the Increase in the Education-Health Association from 1994 to 2019, by U.S. State (Includes Adults of All Employment Statuses)*

41 states in main analysis 9 states not in main analysis
